# Cost-effectiveness of non-communicable disease prevention in Southeast Asia: a scoping review

**DOI:** 10.3389/fpubh.2023.1206213

**Published:** 2023-11-09

**Authors:** Thi-Phuong-Lan Nguyen, M. Rifqi Rokhman, Imre Stiensma, Rachmadianti Sukma Hanifa, The Due Ong, Maarten J. Postma, Jurjen van der Schans

**Affiliations:** ^1^Faculty of Public Health, Thai Nguyen University of Medicine and Pharmacy, Thái Nguyên, Vietnam; ^2^Unit of Global Health, Department of Health Sciences, University Medical Center Groningen, Groningen, Netherlands; ^3^Faculty of Pharmacy, Universitas Gadjah Mada, Groningen, Indonesia; ^4^Unit of Global Health, Department of Health Sciences, University of Groningen, University Medical Center Groningen, Groningen, Netherlands; ^5^Department of Health Financing and Health Technology Assessment, Health Strategy and Policy Institute, Hanoi, Vietnam; ^6^Centre of Excellence in Higher Education for Pharmaceutical Care Innovation, Universitas Padjadjaran, Bandung, Indonesia; ^7^Department of Economics, Econometrics, and Finance, University of Groningen, Groningen, Netherlands; ^8^Department of Economics, Econometrics and Finance, Faculty of Economics and Business, University of Groningen, Groningen, Netherlands; ^9^Faculty of Management Sciences, Open University, Heerlen, Netherlands

**Keywords:** cost-effectiveness, non-communicable disease, prevention, risk factor, Southeast Asia, scoping review

## Abstract

**Background:**

Cost-effectiveness analyses (CEAs) on prevention of non-communicable diseases (NCDs) are necessary to guide decision makers to allocate scarce healthcare resource, especially in Southeast Asia (SEA), where many low- and middle-income countries (LMICs) are in the process of scaling-up preventive interventions. This scoping review aims to summarize the cost-effectiveness evidence of primary, secondary, or tertiary prevention of type 2 diabetes mellitus (T2DM) and cardiovascular diseases (CVDs) as well as of major NCDs risk factors in SEA.

**Methods:**

A scoping review was done following the PRISMA checklist for Scoping Reviews. Systematic searches were performed on Cochrane Library, EconLit, PubMed, and Web of Science to identify CEAs which focused on primary, secondary, or tertiary prevention of T2DM, CVDs and major NCDs risk factors with the focus on primary health-care facilities and clinics and conducted in SEA LMICs. Risks of bias of included studies was assessed using the Consensus of Health Economic Criteria list.

**Results:**

This study included 42 CEAs. The interventions ranged from screening and targeting specific groups for T2DM and CVDs to smoking cessation programs, discouragement of smoking or unhealthy diet through taxation, or health education. Most CEAs were model-based and compared to a do-nothing scenario. In CEAs related to tobacco use prevention, the cost-effectiveness of tax increase was confirmed in all related CEAs. Unhealthy diet prevention, mass media campaigns, salt-reduction strategies, and tax increases on sugar-sweetened beverages were shown to be cost-effective in several settings. CVD prevention and treatment of hypertension were found to be the most cost-effective interventions. Regarding T2DM prevention, all assessed screening strategies were cost-effective or even cost-saving, and a few strategies to prevent T2DM complications were found to be cost-effective in certain settings.

**Conclusion:**

This review shows that the cost-effectiveness of preventive strategies in SEA against T2DM, CVDs, and their major NCDs risk factors are heterogenous in both methodology as well as outcome. This review combined with the WHO “best buys” could guide LMICs in SEA in possible interventions to be considered for implementation and upscaling. However, updated and country-specific information is needed to further assess the prioritization of the different healthcare interventions.

**Systematic review registration:**

https://osf.io, identifier: 10.17605/OSF.IO/NPEHT.

## 1. Introduction

Non-communicable diseases (NCDs) such as type 2 diabetes mellitus (T2DM), cardiovascular diseases (CVDs), cancer, and chronic respiratory diseases are the leading causes of death worldwide and therefore constitute an important global health problem (1). Through the past century, the burden of NCDs was concentrated in developed countries, but in recent years, their incidence, burden, and mortality in low- and middle-income countries (LMICs) have escalated (2–5). Globally, NCDs are responsible for more than 40 million lives lost per year in LMICs, accounting for roughly three quarters of global mortality (6). The United Nations (UN) has responded to this situation by prioritizing the reduction of the burden of NCDs as part of the Sustainable Development Goals (7).

In Southeast Asia (SEA), NCDs such as CVDs or T2DM are emerging as a major and growing burden for the public health sector and the economy. CVDs were the leading cause of death in SEA in 2019 (8). Their crude mortality rate in SEA countries, such as Vietnam, Indonesia or Myanmar, was about 300 per 100, 000 populations in 2019 (8). According to the International Diabetes Federation, there are ~90 million Southeast Asians with diabetes (2). From 2019 to 2045, the number of people with diabetes is expected to increase by over 70% in SEA, compared to only 51% globally (9). Consequently, the economic costs of CVDs, diabetes mellitus and associated complications in SEA will increase correspondingly (10).

Diabetes and CVDs are preventable through controlling modifiable behavioral risk factors (for example by managing tobacco use, physical inactivity, unhealthy diet and alcohol consumption), managing metabolic risk factors (such as hypertension, hyperlipidemia) (11) or early treatment. Therefore, in several SEA countries, national policies or regional programs for the primary (prevention of disease occurrence), secondary (early detection of disease), and tertiary (prevention of disease complications) prevention of NCDs are emerging. However, adequate evidence concerning the cost-effectiveness of the regional interventions is absent, since only a very limited number of rigorous evaluations have been done.

In order to tackle the rising costs of NCDs in SEA, the challenge for decision-makers in healthcare is to implement effective interventions at the lowest possible cost and to find the most cost-effective intervention(s) to combat specific diseases. Cost-effectiveness analysis (CEA) is a helpful tool to prioritize health interventions that will yield the greatest benefits under restricted budgets. This information is essential for SEA countries as most of them are in the process of scaling-up interventions in the course of the “Global strategy for the prevention and control of non-communicable diseases,” which was adopted by the World Health Assembly in 2000 (12).

Therefore, this study aims to review the cost-effectiveness of interventions aimed at primary, secondary and tertiary prevention in LMICs in SEA, that focus on T2DM and CVDs by providing screening and prevention of the main risk factors through targeting people at risk for specific diseases, or who already have those diseases.

## 2. Methods

We provide a review of CEAs of implemented interventions that ranged from prevention and behavior change to screening, diagnostic and care and medical treatment. Interventions had to focus on T2DM and CVDs and the risk factors associated with those diseases, including behavioral risk factors (smoking, alcohol consumption, physical inactivity, and unhealthy diets) and metabolic risk factors (hypertension, hyperlipidemia). The Preferred Reporting Items for Systematic reviews and Meta-Analyses for Scoping Review (PRISMA-ScR) statement was followed for this review (13).

The selection of studies followed the PICO: population: any population within the SEA and must be a low- and middle-income country; Intervention: interventions on type 2 diabetes, cardiovascular diseases and the risk factors associated with those diseases, including behavioral risk factors and metabolic risk factors; Comparator: no limitation on comparator; and Outcome: incremental cost-effectiveness ratio (ICER) or reported both costs and effects. The protocol of this scoping review was registered on the Open Science Framework with the document number 10.17605/OSF.IO/NPEHT.

### 2.1. Search strategy

The search was conducted using the databases *Cochrane Library, EconLit, PubMed, and Web of Science*, for articles published between 01/01/2000 and 30/01/2023. The following search terms were used in combination and modified according to the requirements of the specific database: (T2DM, CVDs and major risk factors) AND (South-East Asia) AND [(community) or (primary healthcare)] and [(intervention) or (evaluation)] AND [(effectiveness) or (cost-effectiveness)]. A detailed example of the complete search terms is presented in Supplementary Document 1.

The titles and abstracts were screened independently by three researchers (TPL Nguyen, JvdS, MRR) to decide on the relevance of each study, and assessed according to predefined inclusion and exclusion criteria (see below). Discrepancies on the inclusion of articles were resolved through discussion followed by mutual consensus between the three researchers to reach a final decision. Next, relevant studies were retrieved in full text and reviewed by the same three researchers. All references of the included articles were scanned for the identification of further articles.

### 2.2. In- and exclusion criteria

We included CEA which focused on primary, secondary, or tertiary prevention of diabetes and CVDs and major risk factors; interventions implemented at primary health-care facilities and clinics as well as at various sites within communities, schools, work sites, and individual homes in a LMIC in SEA. In terms of design, CEA had to be done either in trial-based or model-based design. We excluded CEAs conducted in Singapore, since Singapore is a high-income country in SEA (14). The classification of countries by income is based on the system provided by the UN, which categorizes countries into different income groups based on their Gross National Income per capita. Given the native and learned languages of the research team, studies written in a language that was not English, Burmese, Indonesian or Vietnamese, and studies which were not written as a full original research article in a peer-reviewed journal were also excluded.

### 2.3. Data extraction

Data extraction of each included article was done independently by two researchers, using a custom-made data extraction form in Excel. Discrepancies between the two researchers on the data extraction were resolved through discussion followed by mutual consensus between researchers to reach a final decision. If no consensus was reached, a third author was consulted. The following variables were extracted: disease indication/risk factor, type of intervention, country, design, method, intervention, comparator, population, time horizon, discount rate, currency (reference year), incremental quality-adjusted life years (QALYs)/ life years gained/disability-adjusted life years (DALYs) averted, cost of intervention, cost of the comparator, average cost-effectiveness ratio (ACER), and incremental cost-effectiveness ratio (ICER). If necessary, data were calculated based on the available information provided in the article.

### 2.4. Risk of bias

We assessed the risk of bias by rating each of the included studies using the Consensus of Health Economic Criteria (CHEC)-list (15). The evaluation was conducted by two independent researchers and any disagreement was resolved by the researchers together.

## 3. Results

**Figure 1:**
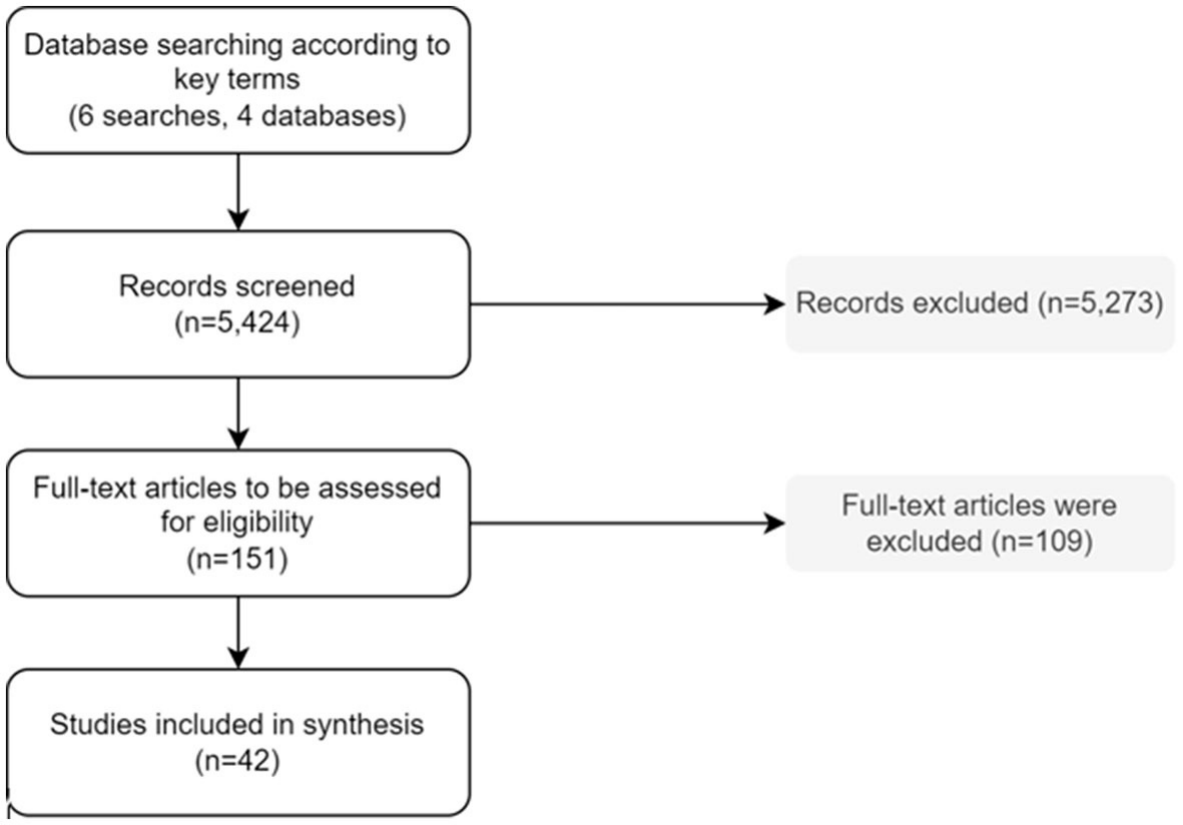
Study selection process.

**Figure 2:**
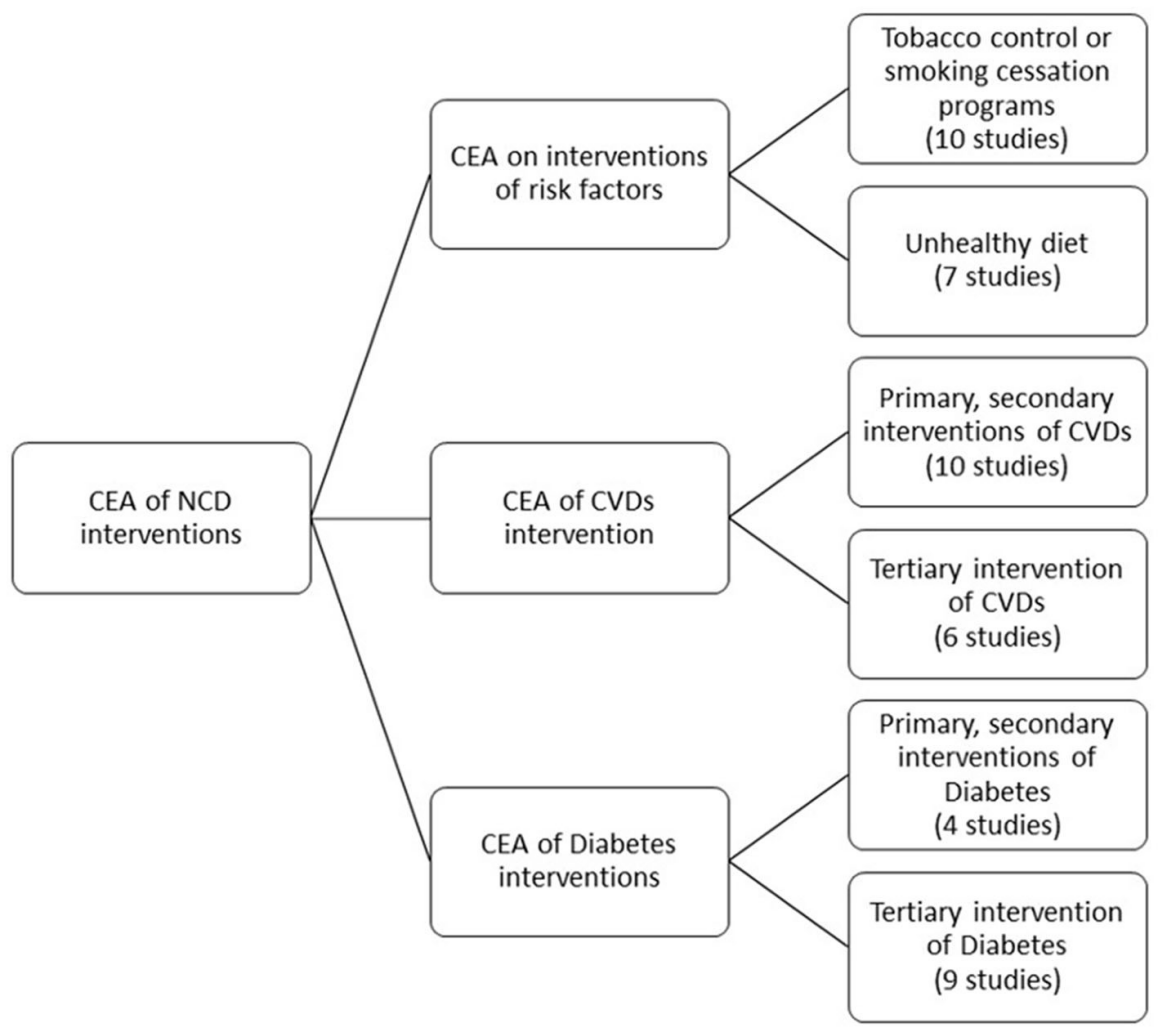
Classification of interventions.

In our scoping review, we included 42 CEAs comparing one or more interventions (Figure 1), consisting of individual interventions, community-based interventions, and/or population-based interventions. The interventions ranged from screening and targeting specific groups of the population for CVD (16–30) and T2DM (high-risk) individuals (16, 31–41) to smoking cessation programs (42–47); or discouragement of smoking or an unhealthy diet through taxation (38, 39, 42, 46, 48–51); or health education (16, 20, 46, 49–52) (Figure 2). We found no CEA that focused on the harmful use of alcohol or physical inactivity.

Almost all studies were based on a cost-effectiveness decision modeling analysis in which a combination of input parameter sources or extrapolation was used to compare the cost-effectiveness of the different interventions. The remaining studies only estimated costs and effects of interventions based on one study (34, 41). Furthermore, the evaluated studies were conducted in single countries (Thailand, Malaysia, Vietnam, Philippines, Indonesia, Myanmar and Cambodia), except the study by Webb conducted in 183 nations which included 3 countries in LMICs in SEA (52).

Table 1 provides an overview of the characteristics and design of each selected study. Most of the studies compared the interventions with a do-nothing scenario, i.e., the cost and health benefits in the absence of the proposed intervention. Furthermore, the minimum of a 10-year implementation horizon was considered in the majority of the studies selected, except for a few studies: Priyadi et al. (34) conducted an observational study over 4 years, Aziz et al. (25) conducted an RCT over 6 months, Satyana et al. (44) modeled participants aged 15–54 years and followed them until 55 years old (44), Hnit et al. (41) only measured one time screening (41), and Nguyen-Thi et al. (40) conducted a modeling study over 5 years (40). In almost all studies, future costs and health benefits were discounted according to the suggested 3% rate, except in the study of Cheng and Estrada (48), which discounted at 7% and in the study of Satyana et al. (44), which discounted at 5%. Four studies did not mention the discount rate at all (25, 32, 39, 54), while the studies by Hnit et al. (41) and Priyadi et al. (34) did not apply discounting. The model-based studies covered interventions in Indonesia, Vietnam, Thailand, the Philippines, Cambodia, Myanmar, and Malaysia.

**Table 1:** Design of costs and effects; cost-effectiveness studies focused on screening, prevention, and/or treatment of diabetes, CVDs or related risk factors.

**Study**	**Disease indication/ Risk factor**	**Type ofintervention**	**Country**	**Design**	**Method**	**Intervention**	**Comparator**	**Population**	**Time horizon**	**Perspective**	**Discount rate**	**Currency (y)**
Thavorn and Chaiyakunapruk (47)	Smoking cessation intervention	Prevention	Thailand	Decision tree and Markov state transition model	CEA	Community pharmacist-based smoking cessation program	Usual care	Population aged 40 who regularly smoke 10–20 cigarettes per day	Lifetime	Health System perspective (direct medical costs)	3%	Thai Baht (2005)
Ha and Chisholm (20)	Cardiovascular disease and risk factors (salt intake, smoking, cholesterol levels)	Prevention and treatment	Vietnam	Population-based simulation model	CUA	Health education through mass media: 1. Reduce salt intake 2. Reduce smoking 3. Reduce cholesterol concentrations 4. Combined strategy (1–3) Individual treatment 5. β-blocker and diuretic for high systolic blood pressure. 6. Statins for high cholesterol concentrations 7. β-blocker, diuretic, statins, and aspirin for individuals with an absolute risk of a cardiovascular event (5%, 15%, 25%, 35% risk).	Null scenario	Vietnamese population (model)	Lifetime	Program and patient-related costs	3%	Vietnamese Dong (2007) (US$1 = VND 16 421 for the base year 2007)
Higashi et al. (53)	Smoking	Prevention	Vietnam	Multi-state life table model (microsimulation dynamic Markov model)	CUA	1. Tax increase on cigarette prices 2. Graphic warning labels on cigarette packs 3. Mass media campaigns against smoking. 4. Expansion of smoking bans to all public places or workplaces	Null scenario	Vietnamese population aged ≥15 years	Lifetime	Governmental perspective (Tax revenue, program costs)	3%	Vietnamese Dong (VND) (2006)
Higashi and Barendregt (42)	Smoking	Prevention	Vietnam	Multi-state life table model (microsimulation dynamic Markov model)	CUA	1. Physician brief advice 2. Nicotine replacement therapy (NRT) patch 3.NRT gum 4.bupropion 5.varenicline.	Null scenario	Vietnamese population aged ≥15 years	Lifetime	Health care perspective	3%	Vietnamese Dong (VND) (2006)
Selvarajah et al. (54)	Hypertension	Screening	Malaysia	Population-based modeling study	CEA	1. Universal screening (aged 30 and above); 2. Those aged 35 and above; 3. Those aged 40 and above; 4. Those aged 45 and above; 5. Those aged 50 and above	<Age 50	Population aged 30 to 74	10 years	Screening costs	Not mentioned	Malaysian Ringgit (US$)
Home et al. (31)	Type 2 diabetes	Treatment	Indonesia (and other countries)	IMS CORE model	CUA	Insulin detemir	Not starting the insulin in people with T2D inadequately controlled on oral glucose-lowering drugs	Insulin-naïve population of Indonesia (model)	30 years	Healthcare perspective	3%	IDR/US$ (2013)
Shafie et al. (27)	Type 2 diabetes	Treatment	Indonesia (and other countries)	IMS CORE model	CUA	Biphasic insulin aspart 30	Not starting biphasic insulin aspart 30 among patients with Inadequately controlled on oral glucose-lowering drugs (oral glucose-lowering drugs)	Insulin-naïve population of Indonesia (model)	30 years	Healthcare perspective	3%	IDR/US$ (2013)
Gupta et al. (32)	Type 2 diabetes	Treatment	Indonesia (and other countries)	IMS CORE model	CUA	Biphasic insulin aspart 30	Biphasic human insulin 30, insulin glargine, or neutral protamine Hagedorn	Indonesian population (model)	30 years	Healthcare perspective	Not mentioned	IDR/US$ (2013)
Nguyen et al. (18)	Hypertension	Screening, prevention	Vietnam	Decision tree and Markov state transition model	CUA	1. No screening 2. One-off screening 3. Screening every 2 years 4. Annual screening 5. Screening in combination with increased coverage of treatment in both sexes and different ages. Various intervals for screening and varying ages to start screening	No screening	Vietnamese population	10 years	Health service perspective (direct medical costs)	3%	I$ (2013)
Permsuwan et al. (55)	Type 2 diabetes	Treatment	Thailand	IMS CORE model	CUA	Insulin Glargine	Neutral protima hagedorn insuline	Thai DM2 population (model)	50 years	Healthcare perspective	3%	Thai Baht/US$ (2014)
Sakulsupsiri et al. (56)	Metabolic syndrome	Prevention	Thailand	Markov state transition model	CUA	Healthy lifestyle persistence of a self-management program	General advice or ordinary care, such as weight control and exercise	Patients with Metabolic syndrome	Lifetime	Societal perspective: (program costs, investment development of program, reinvestment every 5 years).	3%	Thai Baht (2014)
Rattanavipapong et al. (16)	Diabetes, hypertension, and diabetes with hypertension	Screening	Indonesia	Decision tree and Markov state transition model	CUA	1. Current policy (PEN), with random capillary blood glucose screening 2. Policy option 1, screening of individuals aged ≥40 years with fasting capillary blood glucose screening at Posbindu 3. Policy option 2, screening of individuals aged ≥40 years with fasting plasma glucose screening at Puskesmas	No screening	Indonesian population aged ≥15 years	Lifetime	Societal perspective	3%	Indonesian Rupiah (IDR) (2015)
Tosanguan and Chaiyakunapruk (45)	Clinical smoking cessation interventions	Prevention	Thailand	Decision tree and Markov state transition model	CUA	1. Counseling in hospital 2. phone counseling (Quitline) 3. Hospital counseling + nicotine gum 4. Hospital counseling + nicotine patch 5. Hospital counseling + nortriptyline 6. Hospital counseling + bupropion 7. Hospital counseling + varenicline	No intervention (unassisted cessation)	Individuals aged 40 years who smoke at least 10 cigarettes per day	Lifetime	Societal perspective	3%	Thai Baht (2009)
Permsuwan et al. (33)	Type 2 diabetes	Treatment	Thailand	IMS CORE model	CUA	Insulin detemir	Insulin glargine	Thai DM2 population (model)	50 years	Payer's perspective	3%	Thai Baht/US$ (2015)
Webb et al. (52)	Salt intake	Prevention	183 different countries	Global modeling study	CUA	A “soft regulation” national policy that combines targeted industry agreements, government monitoring and public education to reduce population sodium intake.	Null scenario	Full adult population in each country.	10 years	Governmental intervention costs	3%	ppp I$
Bourke and Veerman (38)	Sugar-sweetened beverages	Prevention	Indonesia	Population-based simulation model	CEA	$0.30 per liter tax on sugar-sweetened beverages	No tax	Indonesian population (model)	Lifetime	Governmental perspective (Tax revenue)	3%	I$ (2013)
Tan et al. (43)	Smoking	Prevention	Malaysia	modeling	Estimating costs, effects	Assumption compare smoke and never smoke	Never smoke	Male smokers, aged 15–64 years	Until 65 years old or death	Society (exactly they calculated “productivity-adjusted life years”)	3%	RM and converted to US$
Saxena et al. (39)	Sugar-sweetened beverages	Prevention	Philippines	Mathematical model of disease incidence	CEA	13% tax increase of sugar-sweetened beverages	Null scenario	Philippines population	20 years	Tax revenues, out-of-pocket payments, health care savings	Not mentioned	Philippine pesos (2015)
Dwiprahasto et al. (22)	CVD (Atrial fibrillation, stroke)	Treatment	Indonesia	Markov model	CUA	Treating Atrial fibrillation patients with rivaroxaban for the prevention of stroke	Warfarin	Patients with stroke in Indonesia at 60 years of age	Lifetime	Payer perspective	3%	Indonesian currency (IDR)
Gandola et al. (23)	Lower blood pressure and cholesterol (potassium and phytosterols)	Prevention	Malaysia	Markov model	CUA	Milk powder product fortified with potassium and phytosterols	Do-nothing option	Malaysia population (35–75-year-old population)	40 years	Governmental perspective	3%	International Dollar (I$)
Rattanachotphanit et al. (24)	CVD (Atrial fibrillation, stroke)	Treatment	Thailand	Markov model	CUA	Direct-acting oral anticoagulants for stroke prevention	Adjusted-dose warfarin	Thai patients with non-valvular atrial fibrillation and a HAS-BLED score of 3	20 years	Societal and payer perspectives	3%	US$
Viratanapanu et al. (37)	Type 2 diabetes mellitus, obese	Treatment	Thailand	Decision tree and Markov model	CUA	Bariatric surgery	Usual care	Thai T2DM population with obese	50 years	Healthcare payer's perspective	3%	Thai baht (THB)
Dilokthornsakul et al. (21)	Stroke	Treatment	Thailand	modeling	CEA	Non-Vitamin K Antagonist Oral Anticoagulants (dabigatran 150 mg and 110 mg twice daily; rivaroxaban 20 mg once daily; apixaban 5 mg twice daily; edoxaban 60 mg and 30 mg once daily)	Warfarin	Patients with non-valvular atrial fibrillation	Lifetime	Societal perspective	3%	US$
Krittayaphong and Permsuwan (17)	CVD (Heart failure with reduced ejection fraction)	Treatment	Thailand	Markov model	CUA	Add-on dapagliflozin treatment in heart failure with reduced ejection fraction	Standard treatment without dapagliflozin treatment	Thai population with heart failure with reduced ejection fraction (65 years old)	Lifetime	Healthcare system perspective	3%	THB and US$
Satyana et al. (44)	Smoking	Prevention	Indonesia	modeling	Estimating costs, effects	Assumption compare smoke and never smoke	Never smoke	Indonesian smokers, aged 15–54 years	Until 55 years old	Society (exactly they calculated “productivity-adjusted life years”)	5%	US$
Abdul Aziz et al. (25)	Post-stroke	Treatment	Malaysia	Pragmatic cluster randomized controlled trial	CUA	Integrated Care Pathway for Post Stroke patients	Usual care	Post-stroke patients who referred for longer term stroke care at community health centers in Malaysia	6 months	Societal perspective	-	Malaysian ringgit (MYR)
Ng et al. (26)	Stroke	Prevention	Thailand	Modeling	CEA	Novel oral anticoagulants (NOACs) and warfarin care bundles (e.g. Genotyping, patient self-testing or self-management)	Usual care	Patients with atrial fibrillation	Lifetime	societal perspective/ health care perspective	3%	US$
Taylor et al. (51)	Salt intake	Prevention	Vietnam	Markov model	CUA	Salt substitution strategies by using potassium chloride to reduce sodium intake	No substitution	Vietnam population (model)	Lifetime	Governmental perspective	3%	Vietnamese dong (VND)
Priyadi et al. (34)	Type 2 diabetes	Treatment	Indonesia	Observational study	CEA	Hospitalized T2DM patients with complications of kidney and PVD	Hospitalized T2DM patients without complication	T2DM patients with complications of kidney and peripheral vascular diseases	4 years	Payer and healthcare provider	3%	Indonesian rupiah (IDR)
Nguyen et al. (46)	Tobacco control	Prevention	Vietnam	Markov model	CUA	Population-based tobacco control interventions, including health promotion and education, smoke-free models, cessation programs, warning on package, marketing bans, and raising tax.	No-intervention scenarios	Vietnam population (model)	10 years	Provider perspective	3%	Vietnamese dong (VND)
Feldhaus et al. (35)	Diabetes-related services	Treatment	Cambodia	Markov model	CUA	Financial coverage for diabetes services through the Health Equity Funds	No effective financial coverage for any diabetes-related services	Cambodia population	45 years	Societal perspective	3%	US$
Toi et al. (36)	Type 2 diabetes	Screening	Vietnam	A hybrid of decision tree and Markov models	CUA	Screening for T2DM (1) at CHS and (2) at DHC	No screening	T2DM population in Vietnam (40 years old)	Lifetime	Governmental and societal perspectives	3%	US$
Cheng and Estrada (48)	Smoking	Prevention	Philippines	A static, a single cohort model	CAE	post-cigarette excise tax reform	pre-cigarette excise tax reform	Smokers and non-smokers	Lifetime	Public payer and societal perspectives	7%	US$
Aminde et al. (49)	Salt reduction	Prevention	Vietnam	modeling	CEA	Assumption compares salt reduction (8 g/day, 7 g/day, and 5 g/day targets)	9.4 grams per day (10.5 g/day in men and 8.3 g/day in women)	≥25 years old	6 years, 11 years, lifetime horizon,	Health care perspective	3%	US$
Angell et al. (19)	CVD	Prevention	Indonesia	modeling	CEA	Technology-enabled screening	Usual care	High risk of CVD	10 years	Payer perspective	3%	US$
Nguyen-Thi et al. (40)	Type 2 diabetes	Treatment	Vietnam	Partitioned survival model	CEA	Gliclazide-based intensive glucose control (IGC)	Standard glucose control	T2DM patients	5 years	Healthcare payer perspective.	3%	US$
Krittayaphong and Permsuwan (57)	Acute Decompensated Heart Failure	Treatment	Thailand	Markov model	CUA	• Sacubitril-valsartan • Nalapril for 2 months, then sacubitrilvalsartan	Enalapril	Hospitalized patients with acute decompensated heart failure	Lifetime	Healthcare system perspective	3%	US$ and THB
Mendoza et al. (28)	Heart failure with reduced ejection fraction	Treatment	Philippines	Makov model	CUA	Dapagliflozin in addition to standard therapy	Standard therapy	Patients with heart failure with reduced ejection fraction	Lifetime	Public healthcare provider's perspective	3%	US$
Rattanavipapong et al. (29)	acute ischaemic stroke	treatment	Thailand	Makov model	CEA	• Patients eligible for intravenous alteplase: Alteplase and Endovascular therapy • Patients not eligible for intravenous alteplase: Endovascular therapy	AlteplaseSupportive care	Stroke patients, aged 65 years	Lifetime	Societal perspective	QALYs: 3% each year with 0–2%; Costs: 3% each year with 0–4%	THB
Hnit et al. (41)	Diabetes	Screening	Myanmar	Cross sectional study	CEA	• Diabetic Foot Screen proforma	biothesiometry	DM2 patients at 18 years old and above	One time measurement	Patients' perspective		US$
Thobari et al. (30)	Acute Coronary Disease	Treatment	Indonesia	Decision tree and Markov state transition model	CEA	• Ticagrelor	Clopidogrel	Acute coronary disease	5 years and lifetime	Unclear_ Hospital perspective	3%	US$
Matheos et al. (50)	Smoking	Prevention	Indonesia	Decision tree and Markov state transition model		• Government-funded varenicline • Smoke-free zones/smoking ban • Add 10% tobacco tax	Current situation	Aged 15 to 84 years	Lifetime	Healthcare system	3%	US$

### 3.1. Cost-effectiveness of interventions on main modifiable behavior risk factors for NCDs

#### 3.1.1. Tobacco use

Studied interventions focused on the prevention of tobacco use by means of increasing the price of tobacco products or tax, making tobacco packaging less appealing, banning the marketing of tobacco products, creating a smoke-free environment, eliminating exposure to second-hand tobacco smoke, smoking cessation programs, and mass media campaigns on the harm of tobacco (Table 2).

**Table 2:** Results of costs and effects; cost-effectiveness studies focused on the prevention of tobacco use.

**Study**	**Incremental QALYs/Life years gained/DALYs averted**	**Cost of intervention**	**Cost of comparator**	**ACER**	**ICER**
Thavorn and Chaiyakunapruk (4)	Life-expectancy increase per person Men: 0.181 years Women: 0.244 years	Incremental lifetime cost per person Men: −17,503.54 baht ($-500) Women: −21,499.75 baht ($-614)	0	-	Cost savings of $ 2,777 per life-year gained for men of age 40 (500 $ per 0.18 life-years saved) and cost savings of $ 2,516 per life-year gained for women of age 40
Ha and Chisholm (20)	Total annual DALYs averted	Total costs per year	0	Most cost-effective:	HBP(>160 mmHg) 1,281,596 VND per DALY
	Mass media campaign:	Mass media campaign:		Mass media campaign:	HBP(>140 mmHg) 12,194,115 VND per DALY
	Salt intake: 45 939 DALYs	Salt intake: 89 billion VND		Salt intake 1 945 002 VND (US$118) /DALY averted	Combination (>25% risk) 13,585,810 VND per DALY
	Smoking: 7250 DALYs	Smoking: 89 billion VND		Individual treatment:	Combination (>15% risk) 17,547,288 VND per DALY
	Cholesterol 36 982 DALYs	Cholesterol:89 billion VND		HBP(>160 mmHg): 1 281 596 VND (US$78) /DALY.	Combination (>5% risk) 30,240,689 VND per DALY
	Combination 75 379 DALYs	Combination: 167 billion VND			
	Individual treatment:	Individual treatment:			
	HBP(>140 mmHg) 256 559 DALYs	HBP(>140 mmHg) 941 billion VND			
	HBP (>160mmHg) 205 329 DALYs	HBP (>160mmHg) 264 billion VND			
	Cholesterol (>5.7 mmol/l) 78 179	Cholesterol (>5.7 mmol/l) 2,460 billion VND			
	Cholesterol (>6.2 mmol/l) 52 392	Cholesterol (>6.2 mmol/l) 1,174 billion VND			
	Combination (>5% risk) 404 684 DALYs	Combination (>5% risk) 4,121 billion VND			
	Combination(>15% risk) 344 868 DALYs	Combination (>15% risk) 2,308 billion VND			
	Combination(>25% risk) 303 714 DALYs	Combination (>25% risk) 1,584 billion VND			
	Combination(>35% risk) 264 716 DALYs	Combination (>35% risk) 1,129 billion VND			
Higashi et al. (53)	Total lifetime DALYs averted (x1000):	Total costs (10 years)	0		Costs per DALY averted: Graphic pack warning label 500 VND
	Graphic pack warning label: 2996 DALYs	Graphic pack warning label 1,492 million VND			Tax increase from 55 to 85% 2,900 VND
	Tax increase from 55 to 85%: 4050 DALYs	Tax increase 11 827 million VND			Tax increase from 55 to 75% 4,200 VND
	Tax increase from 55 to 75%: 2788 DALYs	Mass media campaign 147 559 million VND			Tax increase from 55 to 65% 8,600 VND
	Tax increase from 55 to 65%: 1390 DALYs	Smoking ban (public/work) 213 850 million VND			Smoking ban (public) 67,900 VND
	Smoking ban (public): 3099 DALYs				Mass media campaign 78,300 VND
	Mass media campaign: 1873 DALYs				Smoking ban (work) 336,800 VND
	Smoking ban (work): 637 DALYs				
Higashi and Barendregt (42)	DALYs averted per intervention	Costs per intervention	0	Costs per DALY	Incremental costs per DALY
	1. Physician brief advice: 0.014 DALYs	1. Physician brief advice: 24,700 VND		1. Physician brief advice: 1,742 VND	1. Physician brief advice: 1,742 VND
	2. Nicotine replacement therapy (NRT) patch 0.017 DALYs	2. Nicotine replacement therapy (NRT) patch 4,780,000 VND		2. Nicotine replacement therapy (NRT) patch 227,069VND	2. Nicotine replacement therapy (NRT) patch Dominated
	3. NRT gum 0.011 DALYs	3. NRT gum 1,180,000 VND		3. NRT gum 107,826 VND	3. NRT gum Dominated
	4. Bupropion 0.017 DALYs	4. Bupropion 986,000 VND		4. Bupropion 55,854 VND	4. Bupropion Dominated
	5. Varenicline 0.034 DALYs	5. Varenicline.2,350,000 VND		5. Varenicline 70,018 VND	5. Varenicline. Dominated
	1 + 2: 0.035 DALYs	1 + 2: 4,690,000 VND		1 + 2: 134,202 VND	1 + 2: Dominated
	1 + 3: 0.028 DALYs	1 + 3: 1,180,000 VND		1 + 3: 42,803 VND	1 + 3: Dominated
	1 + 4: 0.036 DALYs	1 + 4: 994,000 VND		1 + 4: 27,760 VND	1 + 4: 44,665 VND
	1 + 5: 0.056 DALYs	1 + 5: 2,360,000 VND		1 + 5: 41,561 VND	1 + 5: 65,628 VND
Tosanguan and Chaiyakunapruk (45)	Lifetime QALYs gained per individual Counseling in hospital 0.08 QALY Phone counseling (Quitline) 0.08 QALY	Mean costs per treatment Counseling in hospital−2,808 baht Phone counseling (Quitline)−3,823 baht	-	-	‘Counseling with nortriptyline' and “counseling with varenicline” were the most cost-effective interventions.
	Hospital counseling +	Hospital counseling +			
	Nicotine gum 0.19 QALY	Nicotine gum−6,127 baht			
	Nicotine patch 0.24 QALY	Nicotine patch−3,680 baht			
	Nortriptyline 0.24 QALY	Nortriptyline−11,530 baht			
	Bupropion 0.28 QALY	Bupropion−9,553 baht			
	Varenicline 0.46 QALY	Varenicline−17,922 baht			
Tan et al. (43)	2,951,958 million PALYs	RM 93,261 (US$ 23,502) per PALY			RM 93,695 (US$ 23,611) per PALY
Satyana et al. (44)	15,616,260 PALYs lost	US$11,765 (IDR168,883,998)/ PALYs			(US$ 11,765) per PALY
Nguyen et al. (46)	Number of DALY averted:	Health education and promotion campaigns: 244,335,408 VND			Health education and promotion campaigns: 135,560 VND/DALYS averted
	Health education and promotion campaigns: 1,802,420	Smoke-free model: 188,934,638 VND			Smoke-free model: 67,709 VND/DALYs averted
	Smoke-free model: 2,790,412	Offer smoking cessation services: 19,721,595 VND			Offer smoking cessation services: 12,508 VND/DALYs averted
	Offer smoking cessation services: 1, 576,774	Graphic health warning on tobacco packaging: 4,228,686 VND			Graphic health warning on tobacco packaging: 1,405 VND/DALYs averted
	Graphic health warning on tobacco packaging: 3,009,474	Bans on advertising, promotion and sponsoring: 18,807,103 VND			Bans on advertising, promotion and sponsoring: 63,595 VND/DALYs averted
	Bans on advertising, promotion and sponsoring: 295,732	Raising tobacco taxes (add specific tax of 1000 VND/pack): 16,854,056 VND			Raising tobacco taxes (add specific tax of 1,000 VND/pack): 2,080 VND/DALYs averted
	Raising tobacco taxes (add specific tax of 1000 VND/pack): 8,101,080	Raising tobacco taxes (add specific tax of 2000 VND/pack): 16,854,056 VND			Raising tobacco taxes (add specific tax of 2,000 VND/pack): 1,766 VND/DALYs averted
	Raising tobacco taxes (add specific tax of 2000 VND/pack): 9,544,791				
Cheng and Estrada (48)					
	Public Payer's perspective: 34,571	1,622,339, 000	1,273,083, 000 US$		−10612.73 US$/DALY
	Societal perspective: 34,571	2,696,205, 000	2,394,740,000 US$		−11,995.09 US$/DALY
Matheos et al. (50)	*Differences between the current situation and varenicline (11.6% reduction of smoking prevalence)*	$2 554 533 783 962	$2 868 426 260 361		Dominant
	Dead:−1 220 763				
	Years of life save: 5 473 958				
	QALY: 11 914 970				
	*Differences between the current situation and varenicline (1.6% reduction of smoking prevalence)*				
	Dead:−169 414	$2 842 195 158 191			Dominant
	Years of life save: 759 662				
	QALY: 1 653 529				
	*Differences between the current situation and a smoking ban*				
	Dead:−356 441				
	Years of life save: 1 598 29	$2 774 621 899 004			Dominant
	QALY: 3 478 960				
	*Differences between the current situation and an additional tobacco tax*				
	Dead:−387 892				
	Years of life save: 1 739 325				
	QALY: 3 785 927	$20 736 502 200 434			Dominant

All reviewed studies addressing these interventions confirmed that a tax increase on tobacco products would be cost-effective in the SEA population (46, 48, 50, 53). Higashi et al. (53) concluded that graphic warning labels on cigarette packs would be the most cost-effective option, followed by a tax increase on tobacco products and mass media campaigns to educate about tobacco harm. Furthermore, Nguyen et al. (46) identified that offering smoking cessation services, banning advertising, promotion and sponsoring, and creating smoke-free environments are cost-effective. Ha and Chisholm (20) also concluded that media campaigns against smoking would be very cost-effective in the Vietnamese population (20).

Seven studies assessed the economic and health impact of smoking cessation programs in the SEA setting (42–47, 50). Three of them considered brief advice by a physician and counseling in hospital to be cost-effective (42, 45, 47). However, the study in Vietnam (42) found no cost-effectiveness of physician brief advice compared with pharmaceutical aids, while another study in the Thailand context found the combination of counseling and pharmaceuticals to be cost-effective (45). In the study of Thavorn and Chaiyakunapruk (47) in Thailand, a structured community pharmacist-based smoking cessation program was cost-saving and health gaining compared to usual care.

Moreover, two studies analyzed an intervention that was not considered in the WHO “best buys,” concerning a smoking ban enforced either in public or at work (50, 53). In Indonesia, the smoking ban was dominant compared to the current situation (50). Studies by Tan et al. (43) in Malaysia and Satyana et al. (44) concluded that optimization of smoking cessation programs among those of working age was potentially cost saving in the long term. Yet, these studies did not introduce specific interventions, but based the analysis on assumptions comparing smokers and never-smokers (43, 44). Although smoking cessation was also proven to be cost-effective in the Vietnamese population, the effect was less cost-effective compared to graphic packaging warning labels and taxation of tobacco products (53).

#### 3.1.2. Unhealthy diet

The reduction of sodium/salt intake was recognized as one of the important interventions to control blood pressure and manage CVD events. These salt intake reductions are established by setting a target salt level in foods, providing lower sodium options, communication and media campaigns focused on reducing salt intake or raising awareness through labeling, and setting up a national policy that combines government-industry agreements, government monitoring and public education (20, 49, 51, 52). The reduction of cholesterol levels by medication (20), and reduction of sugar consumption through taxation on sugar-sweetened beverages (38, 39) were also reported to reduce the burden of CVDs (Table 3).

**Table 3:** Results of costs and effects; cost-effectiveness studies focused on prevention of unhealthy diet.

**Study**	**Incremental QALYs/LYs gained/DALYs averted**	**Cost of intervention**	**Cost of comparator**	**ACER**	**ICER**
Ha et al. (20)	Total annual DALYs averted Mass media campaign: Salt intake: 45,939 DALYs Smoking: 7,250 DALYs Cholesterol: 36,982 DALYs Combination: 75,379 DALYs Individual treatment: HBP(>140 mmHg): 256,559 DALYs HBP(>160 mmHg): 205,329 DALYs Cholesterol (>5.7 mmol/l) 78,179 Cholesterol (>6.2 mmol/l) 52,392 Combination (>5% risk) 404,684 DALYs Combination (>15% risk) 344,868 DALYs Combination (>25% risk) 303,714 DALYs Combination (>35% risk) 264,716 DALYs	Total costs per year Mass media campaign: Salt intake: 89 billion VND Smoking: 89 billion VND Cholesterol:89 billion VND Combination: 167 billion VND Individual treatment: HBP(>140 mmHg) 941 billion VND HBP(>160 mmHg) 264 billion VND Cholesterol (>5.7 mmol/l) 2,460 billion VND Cholesterol (>6.2 mmol/l) 1,174 billion VND Combination (>5% risk) 4,121 billion VND Combination (>15% risk) 2,308 billion VND Combination (>25% risk) 1,584 billion VND Combination (>35% risk) 1,129 billion VND	0	Most cost-effective: Mass media campaign: Salt intake 1,945,002 VND (US$118)/DALY averted Individual treatment: HBP(>160 mmHg): 1,281,596 VND (US$78)/DALY.	HBP(>160 mmHg) 1,281,596 VND per DALY HBP(>140 mmHg) 12,194,115 VND per DALY Combination (>25% risk) 13,585,810 VND per DALY Combination (>15% risk) 17,547,288 VND per DALY Combination (>5% risk) 30,240,689 VND per DALY
Webb et al. (52)	Total lifetime DALYs averted Indonesia: 987,857 DALYs Myanmar: 246,217 DALYs Thailand: 270,884 DALYs Vietnam: 246,143 DALYs	Cost per capita (10 years) Indonesia I$0.54 Myanmar I$0.31 Thailand I$0.33 Vietnam I$0.31	0		East / Southeast Asia I$ 123/DALY; Indonesia I$71.48/DALY; Myanmar I$33.30/DALY; Thailand I$ 54.46/DALY; Vietnam I$62.00/DALY
Bourke and Veerman (38)	Total lifetime HALYs gained in population (lowest - highest income quintile): female: 38,382–800,609; male: 30,594–886,920	Revenue tax paid over 25 years (lowest - highest income quintile): $0.5–$15.1 billion	0	-	-
Saxena et al. (39)	No. of diabetes mellitus incident cases averted 299,540 No. of diabetes mellitus deaths averted over 20 years 5,913 No. of ischemic heart disease incident cases averted 40,882 No. of ischemic heart disease deaths averted over 20 years 10,339 No. of stroke incident cases averted 19,858 No. of stroke deaths averted over 20 years 7,950	Total health-care savings over 20 years, billion Philippine pesos 31.6 The total reduction in out-of-pocket payments over 20 years, billion Philippine pesos 18.6 Changes in annual tax revenues, billion Philippine pesos 41.0	-	-	-
Gandola et al. (23)	The milk powder fortified with potassium and phytosterols would help prevent at least: 13,400 MI (−7%), 30,500 strokes (−20%), more than 10,600 MI-related deaths over 40 years more than 17,100 stroke-related deaths over 40 years		-		I$ 22,518.03 per QALY gained
Taylor et al. (51)	Voluntary strategy: 0.009 QALYs Subsidized strategy: 0.022 QALYs Regulatory strategy: 0.074 QALYs	Voluntary strategy: 1,050,036^đ^ (US$ 45.24) Subsidized strategy: 1,010,292^đ^ (US$ 43.53) Regulatory strategy: 809,951^đ^ (US$ 34.90)	1,053,481^đ^ (US$ 45.39)		All three strategies were dominated.
Aminde, et al. (58)	By 2025: over 56,554 stroke-related health-adjusted life years (HALYs)	Saving over US$ 42.6 million in stroke healthcare costs			
	By 2030: about 206,030 HALYs (for 7 g/day target) and 262,170 HALYs (for 5 g/day target)	Saving over US$ 88.1 million HALYs (for the target of 7 g/day) and US$ 122.3 million in stroke healthcare costs (for the target of 5 g/day)			

đ Vietnamese Dong.

Four studies in our review assessed the cost-effectiveness of reducing salt intake through a mass media campaign in Vietnam (20, 49, 51) or in a combination of SEA countries (52). Ha and Chisholm (20) looked at the introduction of a mass media campaign to reduce salt intake compared to a broad context of health care interventions. The study compared different health education interventions through mass media in a Vietnamese setting, i.e., (1) to reduce salt intake; (2) to reduce smoking; (3) to reduce cholesterol concentrations and (4) a combination of these three strategies. A mass media campaign focused on the reduction of salt intake turned out to be the most cost-effective option with a cost-effectiveness ratio of US$ 118/DALY averted. Webb et al. (52) focused on an intervention that combined targeted industry agreements and public education to decrease population sodium intake. Overall, the study concluded that introducing this ‘soft regulation' intervention would be considered highly cost-effective worldwide, since 99.6% of the countries under study identified a cost-effective ratio of <1 times the gross domestic product (GDP) per capita. The ICER of the combined region of South and SEA was 123 I$/DALY. Taylor et al. (51) compared salt substitution strategies using potassium chloride to reduce sodium intake vs. no substitution. They found that all three strategies, e.g., voluntary strategy (no involvement or coordination from government in the market and food industry, no coordinated mass media campaign), subsidized strategy (a communication and media campaign to drive uptake), and regulatory strategy (no media campaign as compliance was assured through regulation) were cost-effective (51).

Bourke and Veerman (38) and Saxena et al. (39) assessed the cost-effectiveness of a tax increase on sugar-sweetened beverages in Indonesia and the Philippines. According to both studies, the tax increase on sugared drinks would be cost-effective in preventing NCDs such as T2DM, ischemic heart disease, stroke, and obesity. The interventions focused on increasing tax compared to no taxation, in which all tax payments came from the client's pocket, instead of the producer's pocket. In both countries, health effects and reduction of out-of-pocket payments for health care services through increasing tax were greater for higher-income quintiles compared to the lower-income quintiles. Nevertheless, assessing the impact of the taxation from a societal perspective instead of a health care perspective or governmental perspective could change the cost-effectiveness of this intervention by including the higher spending of the consumers.

### 3.2. Cost-effectiveness of interventions on CVD

#### 3.2.1. Primary/secondary prevention (e.g., screening and treatment for risk factors)

Within our review, seven studies assessed the prevention of CVD and related risk factors in Vietnam (18, 20), Thailand (56), Indonesia (16, 19) and Malaysia (23, 54).

Interventions focused on individuals in the study by Ha and Chisholm (20) were divided into two categories: treatments based on elevated levels of cholesterol and systolic blood pressure, and treatments based on the 10-year risk (5, 15, 25, and 35% individual risk) of a CVD event. High cholesterol treatment (>5.7 mmol/l and >6.2 mmol/l) was based on treatment with statins, elevated systolic blood pressure (>140 mmHg or >160 mmHg) was treated with a combination of a β-blocker and a diuretic, and individual risk treatment was based on a combination regime of aspirin, diuretics, β-blockers, and statins. The authors concluded that the individual treatment of systolic blood pressure >160 mmHg would be the most cost-effective intervention (US$78 per DALY), even comparing with population-based mass media strategies. However, with a limited budget for investing in such health care interventions, mass media education on salt intake and a combination of targeting salt intake, cholesterol and tobacco should be considered as the first step in the prevention of CVDs. Treatment for elevated levels of systolic blood pressure or at-risk individuals for CVD could also be considered as cost-effective interventions in this country (20). In Thailand, the authors estimated the cost-effectiveness of a self-management program (joining educational session to get information about metabolic syndrome, metabolic control, and self-management skills) vs. the control group (receiving general advice or ordinary care, such as weight control and exercise) among patients with metabolic syndrome. The intervention was found to be cost-effective and recommended to be applied in health care settings, which can reduce the burden of the metabolic syndrome (56). For Malaysia, the consumption of a milk powder product fortified with potassium (+1050.28 mg/day) and phytosterols (+1200 mg/day) was shown to be cost-effective to lower systolic blood pressure and low-density lipoprotein cholesterol, among 35- to 75-year-olds; the ICER was equal to I$ 22, 518.03 per QALY gained (23).

To detect risk factors and undiagnosed CVDs, four studies considered screening as an intervention in Vietnam (18), Malaysia (54), and Indonesia (16, 19). Selvarajah et al. (54) only considered the cost per high CVD risk detected, without the additional treatment. They concluded that a targeted gender- and age-specific screening compared to a universal screening strategy could contribute to effective allocation of already scarce resources. In Vietnam, the strategy of community screening for undiagnosed and untreated hypertension combined with an increase in concurrent treatment to prevent CVD was evaluated. Compared to a no-screening scenario, screening (selected based on age, sex, or screening interval) in general was considered cost-effective in the prevention and early detection of CVD (18). Similarly, Selvarajah et al. (54) found a significant impact of age, sex, and screening interval; a more beneficial cost-effectiveness ratio resulted when considering an increase in treatment uptake (scenario of uptake of treatment, adherence to treatment, and relative risk reductions for those adhering to treatment). A combination of screening and treatment strategies was assessed in the study by Rattanavipapong et al. (16) in the context of the Package of Essential non-communicable disease (PEN) interventions. A no-screening scenario for hypertension and diabetes was compared to the current PEN interventions, with only a once-in-a-lifetime screening, and two adjusted PEN policy options in which screening of high-risk individuals takes place at either the community level or at the primary healthcare level. As expected, implementation of all interventions dominated (fewer costs, higher health benefits) compared to the no-screening scenario, but the PEN strategy is still considered the most cost-effective option. Additionally, targeting specific high-risk individuals within the PEN strategy could improve the cost-effectiveness of this scenario. Another study in Indonesia, by Angell et al. (19), which assessed from a health system perspective, considered a mobile technology-enabled primary care intervention for CVD risk management (health staff assesses CVD risk using mobile technologies and provides a decision support application on a tablet device, including classification of risk level, consultations if needed, reminding patients to attend follow-up visits, adherence to medicine). It showed that the intervention is cost-effective in comparison with the usual care and it was therefore recommended for application in practice (19).

#### 3.2.2. Tertiary prevention

This section covers reports on drug therapy and counseling for individuals who have had heart failure with reduced ejection fraction, atrial fibrillation, myocardial infarction, stroke and post-stroke (17, 21, 22, 24–26, 28–30, 57) (Supplementary Table S1).

All studies considered stroke prevention (21, 22, 24–26, 29), except one which evaluated treatment for heart failure patients with reduced ejection fraction (17, 28, 30, 57). The majority of these studies showed that the interventions were cost-effective (22, 24–26, 29). For example, Rivaroxaban was found to be cost-effective compared to Warfarin and Aspirin for Stroke Prevention Atrial Fibrillation (SPAF) in the Indonesian setting (22). In the study of Rattanachotphanit et al. (24) on patients with non-valvular atrial fibrillation and a high risk of thrombosis, direct-acting oral anticoagulant treatment was found to be cost-effective from both payer and societal perspectives for stroke prevention. One study in Malaysia used the shared care approach and evaluated the integrated care pathway for post stroke patients. It was implemented to guide primary care teams for incorporating further rehabilitation, and regular screening for post-stroke complications among patients residing at home. This intervention was very cost-effective in comparison with usual care (25). The study of Ng et al. (26) aimed to evaluate the cost-effectiveness of non-vitamin K antagonist oral anticoagulants (NOACs) and warfarin care bundles in patients with atrial fibrillation in Thailand; it showed that patient self-management of warfarin was a highly cost-effective intervention, while a novel oral anticoagulant was unlikely to be cost-effective with regard to stroke prevention (26). Among studies on stroke prevention, only the study on NOAC intervention in patients with atrial fibrillation was not found to be cost-effective (21). The study by Krittayaphong et al. (17), which investigated an add-on dapagliflozin treatment for heart failure patients with reduced ejection fraction, showed that it was a cost-effective treatment. In the study by Rattanavipapong et al. (29), both therapy with Alteplase combined with Endovascular vs. Alteplase and therapy of Endovascular vs. supportive care for acute ischemic stroke showed to be cost-effective interventions in Thailand.

Four studies are related to Acute Coronary Disease or heart failure (17, 28, 30, 57). All of them were found to be cost-effective interventions, except one scenario in the study of Mendoza et al. (28). That study in the Philippines suggested that the intervention is only likely to be cost-effective when add-on dapagliflozin treatment is compared with the standard therapy among heart failure with reduced ejection fraction. Krittayaphong and Permsuwan (17) evaluated treatment for heart failure patients with reduced ejection fraction and showed that add-on dapagliflozin treatment was cost-effective compared with standard therapy. At Thobari et al. (30) found that Ticagrelor was vastly more cost-effective compared to clopidogrel in treatment for acute coronary disease to prevent cardiovascular events in the Indonesian setting. Krittayaphong and Permsuwan (57) reported that treating patients with acute decompensated heart failure with Sacubitril-valsartan was cost- effective when compared to enalapril.

### 3.3. Cost-effectiveness of interventions on T2DM

#### 3.3.1. Primary/secondary prevention of T2DM (screening and treating for risk factors)

From the studies included in this review, two studies focused on screening for T2DM in Indonesia (16) and in Vietnam (36), and one focused on the strategy of lifestyle interventions to prevent the development of T2DM in Thailand (56) (Supplementary Table S2).

As mentioned in Section 3.2.1, the PEN strategy was considered dominant (more effects and cost saving) in the screening for T2DM and hypertension (16). The second screening study considered the scenario of screening at community health stations vs. district health centers for different age groups (36). All scenarios were deemed cost-effective interventions, except screening among the group of people younger than 35 years at both community health stations and district health stations (36). The study on lifestyle modification was based on a self-management program (focused on retention of healthy behaviors using the self-management skills the participants were taught) (56). The self-management program was considered to be cost saving, most likely due to the longer time horizon of the analysis (56).

#### 3.3.2. Tertiary prevention of T2DM

Within the diabetic population, preventive foot care, diabetic retinopathy screening, and effective glycemic control are considered in this section. Nine studies addressed the cost-effectiveness of glycemic control in T2DM, mainly assessing the different formulations of insulin (27, 31–35, 40, 55). There was one study on screening for diabetic peripheral neuropathy (41) and one on cost-effectiveness evaluation of bariatric surgery for morbidly obese patients with diabetes (37).

Switching to biphasic insulin from other glycemic control interventions (32) and starting it in insulin naïve patients (27) was found to be cost-effective in Indonesia. Introduction of long-acting insulin in insulin-naïve individuals resulted in a cost-effective scenario in Indonesia (31). However, in the context of Thailand, treatment with long-acting insulin was not considered cost-effective when compared to treatment with neutral protamine Hagedorn insulin (55). Furthermore, treatment with insulin detemir was not a cost-effective strategy, compared to insulin glargine treatment in Thailand (33). It is noted that all of these studies applied the IMS CORE Diabetes Model for their analysis.

The observational study of Priyadi et al. (34) in Indonesia showed that the cost-effectiveness values of T2DM treatment with complications of kidney and peripheral vascular disease varied between health care provider and payer perspectives. Reducing 1 mg/dL blood glucose in T2DM treatment without kidney complication would require lower cost than in T2DM treatment with complication of Peripheral Vascular Disease (PVD). From the perspective of the payer, ICER of complications of kidney disease was IDR 215.723 per 1 mg/dL blood glucose reduction, while that of complications of peripheral vascular disease was IDR 234.591 per 1 mg/dL blood glucose reduction. From the perspective of the healthcare provider, ICER of complications of kidney disease was IDR 166.289 per 1 mg/dL blood glucose reduction and that of complications of PVD was IDR 681.853 per 1 mg/dL blood glucose reduction.

A study in Vietnam showed that gliclazide-based intensive glucose control was cost-effective compared with standard glucose control, from a healthcare payer perspective (40). The ICER for a 5-year scenario was $1, 764 per LY and $1, 878 per QALY. A study in Cambodia that focused on estimating the burden of T2DM, in term of costs and impacts, demonstrated that coverage for medications would be cost-effective, with $27 per DALY averted (35).

A cross-sectional study comparing screening strategies, diabetic foot screen proforma vs. biothesiometry, found ICER equal to $41.79 per diabetic peripheral neuropathy case detected, among diabetic patients in Myanmar (41). Another study in Thailand performed a cost-effectiveness evaluation of bariatric surgery compared to standard treatment for T2DM control in morbidly obese T2DM patients. The ICER was 26, 907.76 THB/QALY, making it a cost-effective intervention (37).

### 3.4. Risk of bias

The quality of the studies reviewed was assessed using the CHEC-list, which identified several key sources of bias (Supplementary Document 2). Limited generalizability was a major concern, with only 22.2% of studies reporting on how their results could be implemented in other settings. Furthermore, only 35.6% of studies employed a societal perspective as recommended by the WHO CHOICE guidelines for cost-effectiveness analysis. Ethical and distributional issues were also frequently overlooked, with only 37.8% of studies explicitly addressing these concerns. Outcomes valuation and the choice of time horizon were additional sources of bias, with only 42.4 and 57.8% of studies, respectively performing model validity and extrapolation of the result into a life-time horizon. These biases highlight the need for caution when interpreting the results by carefully considering the characteristics of the population, the interventions under study, and the assumptions being made on the model.

## 4. Discussion

This review covers the cost-effectiveness of a range of interventions implemented in LMICs in SEA, including Indonesia, Vietnam, Thailand, the Philippines, Cambodia, Myanmar, and Malaysia. The interventions varied from screening and targeting specific groups for T2DM and CVDs to smoking cessation programs, discouragement of smoking or unhealthy diet through taxation, and health education. In CEAs related to tobacco use prevention, the cost-effectiveness of tax increase was confirmed in all related studies. Unhealthy diet prevention, mass media campaign, salt substitution strategy, and tax increase on sugar-sweetened beverages were also shown to be cost-effective in several settings. In addition, for CVD prevention, treatment of hypertension was found to be the most cost-effective intervention. Regarding T2DM prevention, all assessed screening strategies were cost-effective or even cost-saving, and a few strategies to prevent T2DM complications were found to be cost-effective in certain settings.

The WHO presented an updated list in 2017 of “best buys” interventions to inform policymakers on cost-effectiveness; the list includes recommended interventions focused on the prevention and control of NCDs (59). The interventions focus on both the main risk factors for NCDs (tobacco, harmful use of alcohol, unhealthy diet and physical inactivity) and the four disease areas (CVD, T2DM, cancer and chronic respiratory disease). The interventions presented were selected based on proven effectiveness and a clear link to the global NCD targets. All selected interventions were tested against the WHO average cost-effectiveness threshold of ≤ I$ 100/DALY averted in low and lower middle-income countries. Interventions above the I$ 100/DALY averted threshold, or with cost-effectiveness data not available, were labeled as such (59). Country specific or additional data is needed for these two intervention categories. In this literature review, we have provided an overview of the cost-effectiveness studies performed in SEA to compare interventions aimed at preventing or treating T2DM and/or hypertension and related CVDs. Comparing these studies to the WHO “best buys” interventions will help to prioritize interventions or combinations of interventions for upscaling in the SEA region.

Overall, the evidence on cost-effectiveness of prevention and treatment targeted at T2DM, hypertension, and CVD is scarce in SEA. This point was also mentioned in a similar review in LMICs over the world (58); for the prevention of harmful use of alcohol and physical inactivity, it is even absent. The WHO “best buys” and the literature presented in this review give an indication of interventions that are cost-effective in comparison with the absence of implementation. In general, the WHO “best buys” and the local literature were in line with the cost-effectiveness of the interventions reviewed herein. However, considering the limited health budgets in most SEA countries, funding for interventions must be allocated wisely to ensure maximum impact on health outcomes. Therefore, the budget impact of each intervention needs to be considered to establish a sustainable introduction of the specific interventions. Furthermore, prioritization of possible effective interventions requires country-specific information to assess the incremental cost-effectiveness and added value within the current health care systems and compared to any interventions already in place. Scientific evaluations of the cost-effectiveness of multiple preventive interventions and treatment strategies for T2DM and CVD, combined with country-specific data, could give first insights into these priorities. Studies such as those by Ha and Chisholm (20) and Ortegón et al. (60) help to balance the provision of healthcare with the highest value.

The countries included in this review are diverse with regard to economics, culture, implementation capacity, and health systems. No evidence was found for scaling up these interventions from one country to another country in this region. To scale up and transfer interventions to other countries, it is advised to consider other factors such as health impact, acceptability, sustainability, scalability, multisectoral actions, training needs, and suitability of existing facilities, besides the evidence on cost-effectiveness (59). Furthermore, it is important to put the intervention in the health care context of a country, considering potential obstacles to implementation such as different motivation, less adherence to treatment, different availability, and quality of service.

We reviewed the cost-effectiveness of NCD prevention and treatment programs that focused on T2DM, CVD and their risk factors conducted in LMICs in SEA. This review provides initial evidence that can support the efforts of scaling up interventions in this region.

When focusing on tobacco consumption in a community or primary healthcare setting, it is important to consider that patient-focused interventions like counseling are cost-effective. However, in combination with discouragement of tobacco use (e.g., taxation, warning on package) or increased awareness of the harm of tobacco products, they could even be more cost-effective. This finding was in line with results from a previous study of a review of primary and secondary prevention interventions for cardiovascular disease in all LMICs in the world (58, 61). Furthermore, when focusing on unhealthy diets in a community or primary healthcare setting, the reduction of salt intake, even when compared to tobacco use, is considered to be highly cost-effective. A systematic review of economic evaluations of population-based sodium reduction interventions in all settings (62) or in South Asian countries (61) also showed similar results. This suggests that salt reduction should be a primary target when considering changing unhealthy diets. Unfortunately, no specific community based or primary healthcare-based interventions were evaluated with respect to cost-effectiveness in a SEA setting.

When focusing on the primary or secondary prevention of CVD in a community or primary healthcare setting, individual drug treatment should be one of the priorities, even more in comparison to population-based interventions like mass media campaigns focused on, for example, salt intake. Screening, preferably in the community, can be a cost-effective addition in identifying at risk or undiagnosed CVD patients. This finding was also mentioned in recent reviews of primary or secondary prevention interventions for CVD, T2DM in LMICs (58, 63). However, with limited resources available, investing in mass media education in prevention of CVD should be considered first, because of the lower costs. Furthermore, when focusing on the primary or secondary prevention of T2DM in a community or primary healthcare setting, lifestyle interventions and/or drug treatment should be considered.

An approach of combined interventions (treatment and prevention) and the WHO “best buys” recommendations suggest that combining community-based intervention with primary health care will help to reduce costs and provide synergistic effects to interventions (20) and provide an example of such an intervention of mass media education and treatment for hypertension or lowering cholesterol. Another example is the combination of targeted industry agreements and public education in the reduction of sodium intake (52). Multiple SEA countries, like Vietnam, Indonesia, and Myanmar, stayed well below the WHO threshold of 100 I$/DALY averted with an ICER ranging from 30 to 70 I$/DALY averted; therefore, the combined approach may be recommended for other countries in the region. However, further cost-effectiveness evidence of these combined interventions is needed in a local context to decide on the added value per country.

Concerning the intervention design, no evidence was available on the cost-effectiveness of interventions aimed at the underlying health system to improve NCD management, such as interventions that synergize community-based intervention and health facility intervention vs. usual care or that improve the capacity of the health service. Also, evidence on the cost-effectiveness of interventions that treated hypertension integrated with T2DM is not yet available. From the methodological perspective, several studies conducted CEA by comparing an intervention with a no intervention-scenario, while the WHO CEA guideline advice is to compare the intervention with the current best alternative intervention(s) in place. Therefore, future studies may consider using this as a comparator instead.

ICER and thresholds used varied across the studies. This observation is similar to that in a previous review on lung cancer; it is well-known that the cost-effectiveness of interventions can vary in the local environment of one country to another (64). According to WHO “best buys,” except for tertiary prevention among the T2DM population, a cut-off threshold of I$100 per DALY averted in LMICs should be applied to deem an intervention cost-effective. Papers included in this review applied either a cut-off point in terms of GDP per capita per DALY averted or a specific threshold of 160, 000 BAHT/QALY or 120, 000 BAHT/QALY in Thailand (~10, 000–14, 000 I$ or ½ × GDP per capita per DALY averted). Noticeably, some of them did not quantify their outcome as QALY or DALY and did not introduce a threshold in their study (34, 38, 39, 41, 43, 49, 54). In general, CEAs in SEA show cost-effectiveness and recommend applying these interventions in practice. Exceptions are the studies in Thailand by Permsuwan et al. (55) and Permsuwan et al. (33) that switched insulin from one to another type and the study on stroke prevention by Dilokthornsakul et al. (21). However, specific information is needed per country to assess the ICER and added value in the current health care, comparing to the interventions already in place, or to prioritize between different prevention options.

A strength of this review is the identification of the WHO “best buys” as a guideline of possible interventions to be considered for implementation and upscaling in LMICs in SEA. In addition, several interventions are suggested for inclusion in WHO's list, such as screening and managing CVD and DM2, providing pharmacological therapies for reducing tobacco use, or healthy lifestyle to prevent CVD (Box 1).

Box 1 **Recommendations of cost-effectiveness interventions to beat NCD in LMICs in SEA.**

- Reducing tobacco use.
- Discouragement of tobacco use through taxation, warning on package.
- Counseling, brief advice to smokers.
- Health education to increase awareness of the harm of tobacco products.
  Reducing unhealthy diet.
- Reducing salt intake through a government “soft regulation” strategycombines targeted industry agreements, government monitoring, and public education.
- Reducing salt intake through behaviors change communication andmass media campaigns.
- Reducing sugar consumption through effective taxation onsugar-sweetened beverages.
  Prevention and management of CVD.
- Screening and managing CVDs.
- Individual drug treatment.
- Mass media campaign.
  Manage diabetes.
- Individual drug treatment.
- Lifestyle intervention.

A limitation of this study is that updated and country specific information is scarce. Before scaling up any of the interventions, however, further assessment of the prioritization of the different healthcare interventions is needed. Only one study in Vietnam focused on the prioritization between different prevention options (20). In this review, we found a lack of overall prioritization of interventions, while there are many options for interventions to reduce the NCD burden in the region. In the context of budget scarcity, further evidence should be provided to set priorities and to guide local policymakers. Out of the 42 studies included, 37 were designed as modeling studies. These model-based evaluations require many input parameters for their study's purpose, however, most of them lack local context data and must depend on assumptions. These models could be updated when local data of each country becomes available.

Future studies may consider other interventions which reduce harmful alcohol intake, physical inactivity, or investigate synergies between health facility interventions and community interventions. Moreover, they could consider implementation factors in a specific context, such as acceptability, feasibility, and relevance to current policies of a country.

## 5. Conclusion

This review shows that the cost-effectiveness of preventive strategies in SEA against type 2 diabetes mellitus, cardiovascular diseases (CVDs), and their major NCDs risk factors are heterogenous in both methodology as well as outcome. This review combined with the WHO “best buys” list and could be a guideline of possible interventions to be considered for implementation and upscaling in LMICs in SEA. However, updated and country-specific information is needed to further assess the prioritization of the different healthcare interventions. In addition, several interventions which have not yet been included in the “best buys” list could be proposed to WHO for potential inclusion.

## Data availability statement

The original contributions presented in the study are included in the article/Supplementary material, further inquiries can be directed to the corresponding author.

## Author contributions

T-P-LN contributed to conception, design of the study, and wrote the first draft of manuscript. MRR and JvdS organized the database. All authors performed data analysis and interpretation, wrote sections of the manuscript, contributed to manuscript revision, read, and approved the submitted versions.
