# Supplementary material for: Cost-effectiveness of non-communicable disease prevention in Southeast Asia: a scoping review
Source: Front Public Health. 2023 Nov 9;11:1206213. doi: 10.3389/fpubh.2023.1206213 (PMC10666286; doi:10.3389/fpubh.2023.1206213)
Supplement: Supplementary file 1 [file Table_1.DOCX]

Supplementary Material

Cost-Effectiveness of Non-Communicable Disease Prevention in Southeast Asia: A Scoping Review

Thi-Phuong-Lan Nguyen^1*^, M. Rifqi Rokhman^2,3^, Imre Stiensma^4^, Rachmadianti Sukma Hanifa^4^ , Due Ong The^5^, Maarten J. Postma^4,6,7^, Jurjen van der Schans^4,8,9^

*** Correspondence:** Thi-Phuong-Lan Nguyen: [nguyenthiphuonglan@tnmc.edu.vn](mailto:nguyenthiphuonglan@tnmc.edu.vn) or [ntplan75@gmail.com](mailto:ntplan75@gmail.com)

# Example of Complete Search Term

(Major NCDs and major risk factors) AND (South-East Asia) AND ((community) or (primary healthcare)) and ((intervention) or (evaluation)) AND ((effectiveness) or (cost-effectiveness))

# CHEC List

| *Studies* | *Clearly described study population* | *Clearly described competing alternatives* | *Well-defined research question* | *Appropriate economic study design to the stated objectives* | *Appropriate time horizon to include relevant costs and consequences* | *Appropriate chosen perspective* | *Important and relevant costs are identified* | *Appropriate cost measure in physical units* | *Appropriate cost valuation* | *All important and relevant outcomes are identified* | *Appropriate outcomes measurement* | *Appropriate outcomes valuation* | *Incremental analysis of costs and outcomes of alternatives are performed* | *Appropriate discount for future costs and outcomes* | *Sensitivity analysis for uncertain variables* | *Conclusions follow from the reported data* | *Generalizability of the results are discussed* | *No potential conflict of interest is indicated* | *Ethical and distributional issues are discussed* |
| --- | --- | --- | --- | --- | --- | --- | --- | --- | --- | --- | --- | --- | --- | --- | --- | --- | --- | --- | --- |
| Thavorn et al. (2008) | Yes | Yes | Yes | Yes | Yes | No | Yes | Yes | Yes | Yes | Yes | No | Yes | Yes | Yes | Yes | No | Yes | No |
| Ha and Chisholm (2011) | Yes | Yes | Yes | Yes | Yes | Yes | Yes | Yes | Yes | Yes | Yes | No | Yes | Yes | Yes | No | No | No | No |
| Higashi et al. (2011) | Yes | Yes | Yes | Yes | Yes | Yes | Yes | Yes | Yes | Yes | Yes | Yes | Yes | Yes | Yes | Yes | No | Yes | No |
| Higashi and Barendregt (2011b) | Yes | Yes | Yes | Yes | Yes | Yes | No | Yes | Yes | Yes | Yes | No | Yes | Yes | Yes | Yes | No | Yes | No |
| Selvarajah et al. (2013) | Yes | Yes | Yes | No | No | No | No | Yes | Yes | Yes | Yes | No | Yes | Yes | Yes | Yes | No | Yes | Yes |
| Home et al. (2014) | Yes | Yes | Yes | Yes | Yes | No | Yes | Yes | Yes | Yes | Yes | Yes | Yes | Yes | Yes | Yes | Yes | Yes | No |
| Shafie et al. (2014) | Yes | Yes | Yes | Yes | Yes | No | Yes | Yes | Yes | Yes | Yes | Yes | Yes | Yes | Yes | Yes | No | Yes | No |
| Gupta et al. (2015) | Yes | Yes | Yes | Yes | Yes | No | Yes | Yes | Yes | Yes | Yes | Yes | Yes | No | Yes | Yes | No | Yes | No |
| Nguyen et al. (2015) | Yes | Yes | Yes | Yes | Yes | No | Yes | Yes | Yes | Yes | Yes | No | Yes | Yes | Yes | Yes | No | Yes | Yes |
| Permsuwan et al. (2016) | Yes | Yes | Yes | Yes | Yes | Yes | Yes | Yes | Yes | Yes | Yes | Yes | Yes | Yes | Yes | Yes | Yes | Yes | No |
| Sakulsupsiri et al. (2016) | Yes | Yes | Yes | Yes | Yes | Yes | Yes | Yes | Yes | Yes | Yes | Yes | Yes | Yes | Yes | Yes | No | Yes | Yes |
| Rattanavipapong et al. (2016) | Yes | Yes | Yes | Yes | Yes | Yes | Yes | Yes | Yes | Yes | Yes | Yes | Yes | Yes | Yes | No | No | Yes | Yes |
| Tosanguan et al. (2016) | Yes | Yes | Yes | Yes | Yes | Yes | Yes | Yes | Yes | Yes | Yes | No | Yes | Yes | Yes | Yes | No | Yes | No |
| Permsuwan et al. (2017) | Yes | Yes | Yes | Yes | Yes | Yes | Yes | Yes | Yes | Yes | Yes | Yes | Yes | Yes | Yes | Yes | Yes | Yes | No |
| Webb et al. (2017) | Yes | Yes | Yes | Yes | No | No | Yes | Yes | Yes | Yes | Yes | No | No | Yes | Yes | Yes | No | Yes | Yes |
| Bourke et al. (2018) | Yes | Yes | Yes | Yes | No | No | No | Yes | Yes | No | Yes | No | No | Yes | Yes | Yes | No | Yes | Yes |
| Tan, et al. (2018) | Yes | Yes | Yes | No | Yes | No | No | Yes | Yes | No | Yes | No | No | Yes | Yes | Yes | No | Yes | Yes |
| Saxena et al. (2019) | Yes | No | Yes | Yes | No | No | Yes | Yes | Yes | No | Yes | No | No | No | Yes | Yes | No | Yes | No |
| Dwiprahasto et al. (2019) | Yes | Yes | Yes | Yes | Yes | No | Yes | Yes | Yes | Yes | Yes | No | Yes | Yes | Yes | Yes | No | No | No |
| Gandola et al. (2019) | Yes | Yes | Yes | Yes | No | No | Yes | Yes | Yes | Yes | Yes | No | Yes | Yes | Yes | Yes | No | Yes | Yes |
| Rattanachotphanit et al. (2019) | Yes | Yes | Yes | Yes | No | Yes | Yes | Yes | Yes | Yes | Yes | Yes | Yes | Yes | Yes | Yes | Yes | Yes | No |
| Viratanapanu et al. (2019) | Yes | Yes | Yes | Yes | No | No | Yes | Yes | Yes | Yes | Yes | No | Yes | Yes | Yes | Yes | No | Yes | No |
| Dilokthornsakul, et al. (2019) | Yes | Yes | Yes | Yes | Yes | Yes | Yes | Yes | Yes | Yes | Yes | Yes | Yes | Yes | Yes | Yes | No | Yes | No |
| Krittayaphong et al. (2020) | Yes | Yes | Yes | Yes | Yes | No | Yes | Yes | Yes | Yes | Yes | No | Yes | Yes | Yes | Yes | Yes | Yes | No |
| Satyana, et al. (2020) | Yes | Yes | Yes | No | No | No | Yes | Yes | Yes | Yes | Yes | No | No | Yes | Yes | Yes | No | Yes | No |
| Firzah et al. (2020) | Yes | Yes | Yes | Yes | No | Yes | Yes | Yes | Yes | Yes | Yes | No | Yes | No | Yes | Yes | No | Yes | Yes |
| Ng, et al. (2020) | Yes | Yes | Yes | Yes | Yes | Yes | Yes | Yes | Yes | Yes | Yes | No | Yes | Yes | Yes | Yes | Yes | Yes | No |
| Taylor et al. (2021) | Yes | Yes | Yes | Yes | Yes | No | Yes | Yes | Yes | Yes | Yes | Yes | Yes | Yes | Yes | Yes | No | Yes | Yes |
| Priyadi et al. (2021) | Yes | Yes | Yes | Yes | No | No | Yes | Yes | No | Yes | No | No | Yes | Yes | No | Yes | No | Yes | Yes |
| Nguyen et al. (2021) | Yes | Yes | Yes | Yes | No | No | Yes | Yes | Yes | Yes | Yes | No | Yes | Yes | Yes | Yes | No | Yes | Yes |
| Feldhaus et al. (2021) | Yes | Yes | Yes | Yes | No | Yes | Yes | Yes | Yes | Yes | Yes | No | Yes | Yes | Yes | Yes | No | Yes | Yes |
| Toi et al. (2021) | Yes | Yes | Yes | Yes | Yes | Yes | Yes | Yes | Yes | Yes | Yes | Yes | Yes | Yes | Yes | Yes | Yes | Yes | No |
| Cheng et al. (2021) | Yes | Yes | Yes | Yes | No | Yes | Yes | Yes | Yes | Yes | Yes | No | Yes | Yes | Yes | Yes | No | No | No |
| Aminde, et al. (2021) | Yes | Yes | Yes | Yes | Yes | No | Yes | Yes | Yes | Yes | Yes | Yes | No | Yes | Yes | Yes | No | Yes | No |
| Blake Angell, et al. (2021) | Yes | Yes | Yes | Yes | No | No | Yes | Yes | Yes | Yes | Yes | No | Yes | Yes | Yes | Yes | No | Yes | Yes |
| Mendoza et al. (2021) | Yes | Yes | Yes | Yes | Yes | No | Yes | Yes | Yes | Yes | Yes | Yes | Yes | Yes | Yes | Yes | Yes | No | No` |
| Rattanavipapong, et al. (2022) | Yes | Yes | Yes | Yes | Yes | Yes | Yes | Yes | Yes | Yes | Yes | Yes | Yes | Yes | Yes | Yes | No | No | Yes |
| Thobari et al. (2022) | Yes | Yes | Yes | Yes | Yes | No | No | No | Yes | Yes | Yes | Yes | Yes | Yes | No | Yes | No | No | No |
| Nguyen-Thi et al. (2020) | No | Yes | Yes | Yes | No | No | Yes | Yes | Yes | Yes | Yes | Yes | Yes | Yes | Yes | Yes | No | Yes | No |
| Krittayaphong et al. (2021) | Yes | Yes | Yes | Yes | Yes | No | Yes | Yes | Yes | Yes | Yes | Yes | Yes | Yes | Yes | Yes | Yes | Yes | Yes |
| Win Hnit et al. (2022) | Yes | Yes | Yes | Yes | No | No | Yes | Yes | Yes | Yes | Yes | No | No | No | Yes | No | Yes | Yes | Yes |
| Matheos et al. (2023) | Yes | Yes | Yes | Yes | Yes | No | Yes | Yes | Yes | Yes | Yes | Yes | Yes | Yes | Yes | Yes | No | Yes | No |
