# Supplementary material for: Cost-effectiveness of non-communicable disease prevention in Southeast Asia: a scoping review
Source: Front Public Health. 2023 Nov 9;11:1206213. doi: 10.3389/fpubh.2023.1206213 (PMC10666286; doi:10.3389/fpubh.2023.1206213)
Supplement: Supplementary file 2 [file Table_2.DOCX]

**Cost-effectiveness of non-communicable disease prevention in Southeast Asia: A scoping review**

**Description**

This scoping review will explore the cost-effectiveness of interventions aimed at primary, secondary and tertiary prevention in low- and middle-income countries (LMICs) in Southeast Asia (SEA), that focus on type 2 diabetes mellitus (T2DM) and cardiovascular diseases (CVDs) by providing screening and prevention of the main risk factors through targeting people at risk for specific diseases, or who already have those diseases.

**Study design**

We provide a review of cost-effectiveness analysis implemented within the past 22 years on interventions focused on diabetes, CVDs, and their main behavioural risk factors, such as smoking, alcohol consumption, physical inactivity, and unhealthy diets by considering a broad range of (i) prevention and behaviour change, (ii) screening and diagnostic, (iii) care and medical treatment interventions.

We will use the Preferred Reporting Items for Systematic Reviews and Meta-Analysis extension for Scoping Reviews (PRISMA-ScR) [1]. The quality of the selected papers for full text reading will be assessed by using the Consensus on Health Economic Criteria (CHEC) [2].

**Data collection procedures**

Search strategy and data extraction

The search will be conducted using the databases the databases Cochrane Library, EconLit, PubMed, and Web of Science, for articles published between 01/01/2000 and 30/01/2023. The following search terms will be used in combination and modified according to the requirements of the specific database:

1. Search terms: major NCDs and major risk factors

AND

2. Search terms: South-East Asia

AND

3. Search terms: community or primary healthcare

AND

4. Search terms: intervention, evaluation

AND

5. Search terms: effectiveness, cost-effectiveness

Firstly, the title and abstract will be screened independently by two researchers to decide on the relevance of the study. Secondly, relevant studies will be reviewed full text by a team of researchers and assessed according to our inclusion criteria. Of the included articles, all references will be scanned for the identification of further articles.

Data extraction of each included article will be done on a custom-made data extraction form in Excel, independently by two authors. General information of the publication, methodological characteristics of the study, and outcomes will be collected accordingly. Discrepancies between the two authors on both the inclusion of articles as well as the data extraction will be resolved through discussion followed by mutual consensus between researchers to reach a final decision.

Population, intervention, comparator and outcome (PICO) is presented in Table 1. We will include CEA which focused on primary, secondary, or tertiary prevention of diabetes and CVDs and major risk factors; interventions implemented at primary health-care facilities and clinics as well as at various sites within communities, schools, work sites, and individual homes in a LMIC in SEA. In terms of design, CEA has to be done either in trial-based or model-based design. We will exclude CEAs conducted in Singapore, since Singapore is a high-income country in SEA. The classification of countries by income is based on the system provided by the UN, which categorizes countries into different income groups based on their Gross National Income per capita. Given the native and learned languages of the research team, studies written in a language that was not English, Burmese, Indonesian or Vietnamese, and studies which are not written as a full original research article in a peer-reviewed journal will also be excluded.

Table 1. PICO for this review

| Population | any population within the Southeast Asia and must be a low- and middle-income countries |
| --- | --- |
| Intervention | interventions that on type 2 diabetes, cardiovascular diseases and the risk factors associated with those diseases, including behavioral risk factors and metabolic risk factors |
| Comparator | no limitation on comparator |
| Outcome | incremental cost-effectiveness ratio (ICER) or reported both costs and effects |

**Measured variables**

Following variables are recorded: Disease indication/Risk factor, Type of intervention, Country, Design, Method, Intervention, Comparator, Population, Time horizon, Discount rate (%), Currency (year), Incremental QALYs/LYs gained/DALYs averted, Cost of intervention, Cost of the comparator, average cost-effectiveness ratio (ACER), and incremental cost-effectiveness ratio (ICER). If necessary, data is calculated based on the available information in the included article.

**Statistical models**

The included articles will be categorized based on the diseases (diabetes or CVDs) and prevention levels (primary, secondary, or tertiery preventions).

**Study team and their affiliations**

Thi-Phuong-Lan Nguyen^1*^, M. Rifqi Rokhman^2,3^, Imre Stiensma^4^, Rachmadianti Sukma Hanifa^4^ , Due Ong The^5^, Maarten J. Postma^4,6,7^, Jurjen van der Schans^4,8,9^

^1^ Thai Nguyen University of Medicine and Pharmacy, Vietnam.

^2^ Unit of Global Health, Department of Health Sciences, University Medical Center Groningen, Netherlands.

^3^ Faculty of Pharmacy, Universitas Gadjah Mada, Indonesia.

^4^Unit of Global Health, Department of Health Sciences, University of Groningen, University Medical Center Groningen, Netherlands

^5^Department of Health Financing and Health Technology Assessment, Health Strategy and Policy Institute, Ha Noi, Vietnam.

^6^Centre of Excellence in Higher Education for Pharmaceutical Care Innovation, Universitas Padjadjaran, Bandung, Indonesia

^7^Department of Economics, Econometrics & Finance, University of Groningen, Netherlands

^8^ Department of Economics, Econometrics and Finance, Faculty of Economics & Business, University of Groningen, Netherlands.

^9^ Faculty of Management Sciences, Open University, Netherlands.

**References**

1. Tricco, A.C., et al., *PRISMA extension for scoping reviews (PRISMA-ScR): checklist and explanation.* Annals of internal medicine, 2018. **169**(7): p. 467-473.

2. Evers, S., et al., *Criteria list for assessment of methodological quality of economic evaluations: Consensus on Health Economic Criteria.* International journal of technology assessment in health care, 2005. **21**(2): p. 240-245.
