# Supplementary material for: Cost-effectiveness of non-communicable disease prevention in Southeast Asia: a scoping review
Source: Front Public Health. 2023 Nov 9;11:1206213. doi: 10.3389/fpubh.2023.1206213 (PMC10666286; doi:10.3389/fpubh.2023.1206213)
Supplement: Supplementary file 3 [file Table_3.DOCX]

Supplementary Material

Cost-Effectiveness of Non-Communicable Disease Prevention in Southeast Asia: A Scoping Review

Thi-Phuong-Lan Nguyen^1*^, M. Rifqi Rokhman^2,3^, Imre Stiensma^4^, Rachmadianti Sukma Hanifa^4^ , Due Ong The^5^, Maarten J. Postma^4,6,7^, Jurjen van der Schans^4,8,9^

Corresponding Author
[nguyenthiphuonglan@tnmc.edu.vn](mailto:nguyenthiphuonglan@tnmc.edu.vn); ntplan75@gmail.com

**S1 Table: Results of costs and effects; cost-effectiveness studies focused on screening, prevention, and/or treatment of CVDs**

| **Study** | **Incremental QALYs/LYs gained/DALYs averted** | **Cost of intervention** | **Cost of comparator** | **ACER** | **ICER** |
| --- | --- | --- | --- | --- | --- |
| Ha and Chisholm  (2011) | Total annual DALYs averted  Mass media campaign:  Salt intake: 45 939 DALYs  Smoking: 7250 DALYs  Cholesterol 36 982 DALYs Combination 75 379 DALYs  Individual treatment: HBP(>140mmHg) 256 559 DALYs HBP(>160mmHg) 205 329 DALYs Cholesterol (>5.7 mmol/l) 78 179 Cholesterol (>6.2 mmol/l) 52 392 Combination (>5% risk) 404 684 DALYs Combination(>15% risk) 344 868 DALYs Combination(>25% risk) 303 714 DALYs Combination(>35% risk) 264 716 DALYs | Total costs per year  Mass media campaign:  Salt intake: 89 billion VND Smoking: 89 billion VND Cholesterol:89 billion VND Combination: 167 billion VND  Individual treatment:  HBP(>140mmHg) 941 billion VND HBP(>160mmHg) 264 billion VND Cholesterol (>5.7 mmol/l) 2460 billion VND Cholesterol (>6.2 mmol/l) 1174 billion VND Combination (>5% risk) 4121 billion VND  Combination (>15% risk) 2308 billion VND Combination (>25% risk) 1584 billion VND  Combination (>35% risk) 1129 billion VND | 0 | Most cost-effective:  Mass media campaign:  Salt intake 1,945,002 VND (US$118) /DALY averted  Individual treatment:  HBP(>160mmHg): 1,281,596 VND (US$78) /DALY. | HBP(>160mmHg) 1,281,596 VND per DALY  HBP(>140mmHg) 12,194,115 VND per DALY  Combination (>25% risk) 13,585,810 VND per DALY  Combination (>15% risk) 17,547,288 VND per DALY  Combination (>5% risk) 30,240,689 VND per DALY |
| Selvarajah et al. (2013) | Number needed to screen and treat (10 years) to prevent one cardiovascular event:  > age 50 62.24 people  > age 45 76.14 people  > age 40 96.64 people  >age 35 123.95 people  Universal screening 146.73 people | Cost to detect 1 high risk individual  > age 50: 92.64 MYR ($29.73)  >age 45: 110.08 MYR($35.33)  >age 40: 131.51 MYR ($42.20)  >age 35: 158.13 MYR ($50.75)  Universal screening: 187.20 MYR ($60.08) |  |  | Incremental cost per additional high-risk individual detected compared to >age 50 (USD)  >age 45: 490 MYR ($157)  >age 40: 2,060 MYR ($661)  >age 35: 15,483 MYR ($4,968)  Universal screening: 358,444 MYR ($358,444) |
| Nguyen et al. (2016) | QALYs gained per 1,000 persons (10 years)  Biannual screening alone (starting at age 55, male) 2.039 QALYs  Combined with 20% increased treatment coverage (starting at age 55, male) 2.887 QALYs  Biannual screening alone (starting at age 55, female)1.017 QALYs  Combined with 20% increased treatment 1.344 QALYs | Costs per 1000 persons (10 years)  Biannual screening alone (starting at age 55, male) I$4,234  Combined with 20% increased treatment coverage (starting at age 55, male) I$963  Biannual screening alone (starting at age 55, female) I$11,375  Combined with 20% increased treatment I$9,982 | 0 |  | Biannual screening alone (starting at age 55, male) I$2,076/QALY  Combined with 20% increased treatment coverage I$334/QALY  Biannual screening alone (starting at age 45, female) I$11,189/QALY  Combined with 20% increased treatment I$7,425/QALY |
| Sakulsupsiri et al. (2016) | 0.0098 QALY (12 weeks)  (difference-in-difference between week 0 and week 12 for intervention and control group) | Week 0: 567,600 (512,150–623,740)  Week 12: 563,920 (508,370–621,580) | Week 0: 562,060 (502,000–617,500)  Week 12:  560,680 (501,800–618,860) |  | -2,310 bath (12 weeks)  (Difference-in-difference between week 0 and week 12 for intervention and control group) |
| Rattanavipapong et al. (2016) | Lifetime DALYs averted per individual  Current policy: 0.04 DALYs  Policy option 1: 0.02 DALYs  Policy option 2: 0.03 DALYs | Total costs of intervention  Current policy: 57.86 million IDR  Policy option 1: 57.88 million IDR  Policy option 2: 57.66 million IDR | Total costs of No screening policy: 58.33 million IDR | All interventions dominate the comparator | Incremental costs per DALY averted compared to current policy  Policy option 1: Dominated Policy option 2: Saves costs, less effective |
| Dwiprahasto et al. (2019) | 0.18 QALYs | IDR 47,789,630 | IDR 21,689,839 |  | IDR 141,835,063 per QALY |
| Gandola et al. (2019) | The milk powder fortified with potassium and phytosterols would help prevent at least:  13,400 MI (−7%),  30,500 strokes (−20%),  more than 10,600 MI-related deaths over 40 years,  more than 17,100 stroke-related deaths over 40 years |  | - |  | I$ 22,518.03 per QALY gained |
| Rattanachotphanit et al. (2019) | Societal perspective:  Apixaban 5 mg twice daily: 0.34 QALYs  Rivaroxaban 20 mg once daily: 0.41 QALYs  Edoxaban 30 mg once daily:0.36 QALYS  Edoxaban 60 mg once daily: 0.75 QALYs  Dabigatran 110 mg twice daily: 0.47 QALYs  Dabigatran 150 mg twice daily: 0.68 QALYs  Payer perspective:  Apixaban 5 mg twice daily: 0.34 QALYs  Rivaroxaban 20 mg once daily: 0.41 QALYs  Edoxaban 30 mg once daily:0.36 QALYS  Edoxaban 60 mg once daily: 0.75 QALYs  Dabigatran 110 mg twice daily: 0.47 QALYs  Dabigatran 150 mg twice daily: 0.68 QALYs | Societal perspective:  Apixaban 5 mg twice daily: US$ 11,651  Rivaroxaban 20 mg once daily: US$ 11,686  Edoxaban 30 mg once daily: US$ 11,773  Edoxaban 60 mg once daily: US$ 11,832  Dabigatran 110 mg twice daily: US$ 12,074  Dabigatran 150 mg twice daily: US$ 12,168  Payer perspective:  Apixaban 5 mg twice daily: US$ 10,684  Rivaroxaban 20 mg once daily: US$ 10,690  Edoxaban 30 mg once daily: US$ 10,693  Edoxaban 60 mg once daily: US$ 10,918  Dabigatran 110 mg twice daily: US$ 11,128  Dabigatran 150 mg twice daily: US$ 11,278 | US $ 4,602  US $ 3,602 |  | Apixaban 5 mg twice daily: US$ 20,763 per QALY  Rivaroxaban 20 mg once daily: US$ 507 per QALY  Edoxaban 30 mg once daily: Dominated  Edoxaban 60 mg once daily: US$ 434 per QALY  Dabigatran 110 mg twice daily: Dominated  Dabigatran 150 mg twice daily: Dominated  Payer perspective:  Apixaban 5 mg twice daily: US$ 20,857 per QALY  Rivaroxaban 20 mg once daily: US$ 88 per QALY  Edoxaban 30 mg once daily: Dominated  Edoxaban 60 mg once daily: US$ 677 per QALY  Dabigatran 110 mg twice daily: Dominated  Dabigatran 150 mg twice daily: Dominated |
| Dilokthornsakul et al. (2020) | Dabigatran 150mg: 0.29  Dabigatran 110mg: 0.27  Apixaban 5mg: 0.43  Rivaroxaban 20mg: 0.22  Edoxaban 60mg: 0.33  Edoxaban 30mg: 0.32 | Dabigatran 150mg: 481,658 (THB)  Dabigatran 110mg: 483,996 (THB)  Apixaban 5mg: 458,173 (THB)  Rivaroxaban 20mg: 455,501 (THB)  Edoxaban 60mg: 458,167 (THB)  Edoxaban 30mg: 450,649 (THB) | Warfarin:  158,814 |  | Dabigatran 150mg: 34,811 US$/QALY  Dabigatran 110mg: 37,756 US$/QALY  Apixaban 5mg: 21,862 US$/QALY  Rivaroxaban 20mg: 42,556 US$/QALY  Edoxaban 60mg:28,799 US$/QALY  Edoxaban 30mg: 28,861 US$/QALY  All NOACs were not cost-effective strategies |
| Krittayaphong and Permsuwan (2021a) | 0.60 QALY | 54,405 THB or 1,745USD | 17,442 THB or 559 USD |  | 62,090 THB/QALY (1,991 USD/QALY) |
| Aziz et al. (2020) | 0.23 QALYs | MYR 790.34 | MYR 527.22 |  | MYR 1,144 per QALY |
| Ng et al. (2020) | Societal perspective:  Genotype-guided dosing: 0.03  PSM:0.49  PST:-0.22  Rivaroxaban: 0.31  Apixaban: 0.53  Edoxaban: 0.45  Dabigatran: 0.40  LAAC: 0.57 | Societal perspective (US$)  Genotype-guided dosing: 1,498  PSM:2,109  PST:2,427  Rivaroxaban: 5,806  Apixaban: 6,006  Edoxaban: 6,039  Dabigatran: 6,375  LAAC: 9,404 | Usual care:  1,421 US$ |  | PSM: 1,395 US$/QALY  LAAC: 93,830 US$/QALY  All others are dominated  Self-management vs usual care: 1395US$/QALY; All NOACs vs usual care: Not cost-effective (ICER ranging from USD8,678 to USD14,247/QALY) |
| Angell et al. (2021) | 0.01 DALY averted/ 0.02 CVD events | 143.61 US$ | 83.42 US$ |  | 4,288 US$ / DALY; 3,681 US$ / CVD event |
| Mendoza et al. (2021) | Not available | Not available | Not available |  | 3,638 US$ per QALY |
| Rattanavipapong et.al. (2022) | Patients eligible for intravenous alteplase: 1.44 QALY  Patients not eligible for intravenous alteplase: 1.52 | Patients eligible for intravenous alteplase: THB 848700  Patients not eligible for intravenous alteplase: THB 721300 | Alteplase: 637600  Supportive care: 547000 |  | Patients eligible for intravenous alteplase: 146,800 THB per QALY  Patients not eligible for intravenous alteplase: 115,000 THB per QALY |
| At Thobari et al. (2022) | **Life time:**  Life years: 0.0947  QALYs: 0.0828  **Five years**  Life years: 0.0462  QALYs: 0.0410 | **Life time:** US$ 8407.28  **Five years: US$** 7810.11 | **Life time:** US$ 8301.49  **Five years: US$** 7713.98 |  | **Life time:**  US$ 1117.047 per life year  US$ 1278.361 per QALY  **Five years**  US$ 2081.369 per life year  US$ 2343.269 per QALY |

**S2 Table. Results of cost-effectiveness decision modelling studies focused on screening, prevention, and/or treatment of diabetes.**

| **Study** | **Incremental QALYs/LYs gained/DALYs averted** | **Cost of intervention** | **Cost of comparator** | **ACER** | **ICER** |
| --- | --- | --- | --- | --- | --- |
| Home et al. (2015) | Gained per person within 30 years:  1.83 QALYs  0.61 life years | Costs per person within 30 years  $24,031 | Costs per person within 30 years  $22,600 |  | ICER 30 years:  $415 per QALY |
| Shafie et al. (2014) | 30-year gained per person:  2.73 QALYs  1.8 life years | 30-year costs per person:  US$29,077 | 30-years cost per person:  US$24,275 |  | 30-year ICER: US$1,632/QALY  1-year ICER: 4,206US$/QALY |
| Gupta et al. (2015) | QALY gained per person within 30 years:  BHI to BiAsp 30: 0.83 | Incremental costs per person within 30 years  BHI to BiAsp 30: 42,562,608 IDR | 0 |  | ICER 30 years:  BHI to BiAsp 30: 51,416,659 IDR ($4,603) per QALY |
| Permsuwan et al. (2016) | 488 QALY  677 Life years | 661,439 THB | 541,806 THB |  | 176,525 THB/ life year (5,201.09 US$/ life year)  244,915 THB/QALY (7,216.12US$/QALY) |
| Sakulsupsiri et al. (2016) | Lifetime QALY gained per patient  0.0098 QALY | Lifetime costs per patient (compared week 0 vs week 12)  -3,680 baht | Lifetime costs per patient  -1,370 baht  (compared week 0 vs week 12) |  | ICER dominant among patients with metabolic syndrome |
| Rattanavipapong et al. (2016) | Compare with no screening:  Current policy (PEN) – applying random capillary blood glucose for screening: 0.04 DALY averted  Policy option 1 (using fasting capillary blood glucose for screening): 0.02 DALY averted  Policy option 2 (using fasting plasma glucose for screening): 0.03 DALY averted | Current policy (PEN): 57.86 million IDR  Policy option 1: 57.88 million IDR  Policy option 2: 57.66 million IDR | No screening: 58.33 million IDR |  | Dominance  Dominance  Dominance  (Million IDR per DALY Averted) |
| Permsuwan et al. (2017) | Insuline Glargine: 8.908 QALY; 13.116 Life years  Insuline Determir (mixed dose): 8.921 QALY; 13.119 Life years  Insuline Determir (single dose): 8.921 QALY; 13.119 Life years  Insuline Determir (double dose): 8.921 QALY; 13.119 Life years | Insuline Determir (mixed dose): 90,417.63 US$  Insuline Determir (single dose): 60,645.90 US$  Insuline Determir (double dose): 99,439.28 US$ | Insuline Glargine: 66,674.03 US$ |  | Insuline Determir (mixed dose):62,990,376 BTH/ QALY or 1,745,852.99 US$/QALY  Insuline Determir (single dose): cost saving  Insuline Determir (double dose): 86,924,234 BTH/QALY or  2,409,208.26 US$/QALY |
| Viratanapanu et al. (2019) | 2.83 QALYs | 436,928.72 THB | 360,810.89 THB |  | 26,907.76 THB/QALY |
| Priyadi et al. (2021) | Perspective of payer:  Complication of kidney disease: 14 mg/dL  Complication of PVD: 34 mg/dL  Perspective of healthcare provider:  Complication of kidney disease: 14 mg/dL  Complication of PVD: 34 mg/dL | Perspective of payer:  Complication of kidney disease: IDR 12,484,513  Complication of PVD: IDR 17,431,034  Perspective of healthcare provider:  Complication of kidney disease: IDR 10,874,833  Complication of PVD: IDR 31,472,019 | IDR 9,572,251  IDR: 8,629,930 |  | Perspective of payer:  Complication of kidney disease: IDR 215,723 per 1 mg/dL blood glucose reduction  Complication of PVD: IDR 234,591 per 1 mg/dL blood glucose reduction  Perspective of healthcare provider:  Complication of kidney disease: IDR 166,289 per 1 mg/dL blood glucose reduction  Complication of PVD: IDR 681,853 per 1 mg/dL blood glucose reduction |
| Feldhaus et al. (2021) | HEF eligibility 20%  Current standard: -  Diagnostics only: 9,222 DALYs  Drug therapy only: -38,404 DALYs  Complications only: -  Diagnostics + Drug therapy: -38,341 DALYs  Drug therapy + Complications: -38,403 DALYs  Diagnostics + Drug therapy + Complications: -38,341 DALYs | HEF eligibility 20%  Current standard: US $ 222,241,881  Diagnostics only: US $ 375,570,938  Drug therapy only: US $ 223,283,447  Complications only: US $ 376,546,924  Diagnostics + Drug therapy: US $ 365,140,541  Drug therapy + Complications: US $ 365,438,949  Diagnostics + Drug therapy + Complications: US $ 658,879,070 | US $ 222,241,881 |  | HEF eligibility 20%  Drug therapy only: US $ 27/DALY averted  Diagnostics + Drug therapy: US $ 3,727/DALY averted  Drug therapy + Complications: US $ 3,729/DALY averted  Diagnostics + Drug therapy + Complications: US $ 11,388/ DALY averted |
| Toi et al. (2021) | Societal perspective, screening at CHS:  One-off screening: 0.01 QALY  Annual screening: 0.40 QALY  3-yearly screening: 0.14 QALY  Societal perspective, screening at DHC:  One-off screening: 0.02 QALY  Annual screening: 0.43 QALY  3-yearly screening: 0.17 QALY  Provider perspective, screening at CHS:  One-off screening: 0.01 QALY  Annual screening: 0.40 QALY  3-yearly screening: 0.14 QALY  Provider perspective, screening at DHC:  One-off screening: 0.03 QALY  Annual screening: 0.43 QALY  3-yearly screening: 0.17 QALY | Societal perspective, screening at CHS:  One-off screening: US $ 931.62  Annual screening: US $ 724.49  3-yearly screening: US $ 894.63  Societal perspective, screening at DHC:  One-off screening: US $ 947.26  Annual screening: US $ 815.71  3-yearly screening: US $ 856.87  Provider perspective, screening at CHS:  One-off screening: US $ 223.52  Annual screening: US $ 180.57  3-yearly screening: US $ 216.86  Provider perspective, screening at DHC:  One-off screening: US $ 226.51  Annual screening: US $ 188.05  3-yearly screening: US $ 227.45 | US $ 910.70US $ 910.70  US $ 218.1 |  | Societal perspective, screening at CHS:  One-off screening: US $ 2,077/QALY gained  Annual screening: Dominant  3-yearly screening: Dominant  Societal perspective, screening at DHC:  One-off screening: US $ 2,139/QALY gained  Annual screening: Dominant  3-yearly screening: US $ 268/QALY gained  Provider perspective, screening at CHS:  One-off screening: US $ 539/QALY gained  Annual screening: Dominant  3-yearly screening: Dominant  Provider perspective, screening at DHC:  One-off screening: US $ 493/QALY gained  Annual screening: Dominant  3-yearly screening: US $ 54/QALY gained |
| Nguyen-Thi et al. (2020) | 0.017 life years 0.016 QALYs | US$ 3786 | US$ 3757 |  | $1,764 per LY; $ 1,878 per QALY |
| Hnit et al. (2022) | - 122 case detected | US$ 3080 | US$ 7760 |  | US $ 41.79 per case detected |
